# The neuropeptide calcitonin gene-related peptide alpha is essential for bone healing

**DOI:** 10.1016/j.ebiom.2020.102970

**Published:** 2020-08-24

**Authors:** Jessika Appelt, Anke Baranowsky, Denise Jahn, Timur Yorgan, Paul Köhli, Ellen Otto, Saeed Khomeijani Farahani, Frank Graef, Melanie Fuchs, Aarón Herrera, Michael Amling, Thorsten Schinke, Karl-Heinz Frosch, Georg N. Duda, Serafeim Tsitsilonis, Johannes Keller

**Affiliations:** a Center for Musculoskeletal Surgery, Charité - Universitätsmedizin Berlin, 13353, Berlin, Germany; b Julius Wolff Institute for Biomechanics and Musculoskeletal Regeneration, Charité-Universitätsmedizin Berlin, Berlin 13353, Germany; c Department of Trauma and Orthopedic Surgery, University Medical Center Hamburg-Eppendorf, Hamburg 20246, Germany; d Department of Osteology and Biomechanics, University Medical Center Hamburg Eppendorf, Hamburg 20246, Germany

**Keywords:** Fracture, Bone regeneration, Osteoblasts, Osteoclasts, Neuropeptides, αCGRP, CRLR, Olcegepant

## Abstract

**Background:**

Impaired fracture healing represents an ongoing clinical challenge, as treatment options remain limited. Calcitonin gene-related peptide (CGRP), a neuropeptide targeted by emerging anti-migraine drugs, is also expressed in sensory nerve fibres innervating bone tissue.

**Method:**

Bone healing following a femoral osteotomy stabilized with an external fixator was analysed over 21 days in αCGRP-deficient and WT mice. Bone regeneration was evaluated by serum analysis, µCT analysis, histomorphometry and genome-wide expression analysis. Bone-marrow-derived osteoblasts and osteoclasts, as well as the CGRP antagonist olcegepant were employed for mechanistic studies.

**Findings:**

WT mice with a femoral fracture display increased CGRP serum levels. αCGRP mRNA expression after skeletal injury is exclusively induced in callus tissue, but not in other organs. On protein level, CGRP and its receptor, calcitonin receptor-like receptor (CRLR) complexing with RAMP1, are differentially expressed in the callus during bone regeneration. On the other hand, αCGRP-deficient mice display profoundly impaired bone regeneration characterised by a striking reduction in the number of bone-forming osteoblasts and a high rate of incomplete callus bridging and non-union. As assessed by genome-wide expression analysis, CGRP induces the expression of specific genes linked to ossification, bone remodeling and adipogenesis. This suggests that CGRP receptor-dependent PPARγ signaling plays a central role in fracture healing.

**Interpretation:**

This study demonstrates an essential role of αCGRP in orchestrating callus formation and identifies CGRP receptor agonism as a potential approach to stimulate bone regeneration. Moreover, as novel agents blocking CGRP or its receptor CRLR are currently introduced clinically for the treatment of migraine disorders, their potential negative impact on bone regeneration warrants clinical investigation.

**Funding:**

This work was funded by grants from the Else-Kröner-Fresenius-Stiftung (EKFS), the Deutsche Forschungsgemeinschaft (DFG), and the Berlin Institute of Health (BIH).

**Research in context**

**Evidence before this study**

CGRP represents a neuropeptide which is crucially involved in the pathogenesis of migraine, a neurologic disorder affecting almost 15% of the worldwide population. Novel drugs inhibiting CGRP signaling are currently introduced clinically for the prevention of migraine. However, concerns have been raised about hitherto unrecognized adverse effects in extracranial tissues. In skeletal tissue, sensory fibres expressing CGRP innervate bone, yet their role in bone regeneration following fracture is unclear. Increased CGRP levels have been observed in patients with long-bone fractures as well as intense in-growth of new nerve fibres containing CGRP at the fracture site in rats. Although inactivation of αCGRP was suggested to alter macrophage polarization without affecting callus maturation in ovariectomized mice, it was also demonstrated that magnesium implants promote bone regeneration in rats through activation of the CGRP receptor, comprised of calcitonin receptor-like receptor (CRLR) and RAMP1.

**Added value of this study**

In this study, we demonstrate an essential role of αCGRP in orchestrating callus formation and bone regeneration. Our data suggest that CGRP receptor agonism may be a suitable approach to stimulate fracture healing in patients with impaired bone regeneration. Furthermore, they indicate that novel CGRP blockers may negatively affect fracture repair.

**Implications of all the available evidence**

Our results highlight the importance of αCGRP as a pivotal driver of bone regeneration. As CGRP is primarily expressed in innervating sensory fibres, the study additionally emphasizes the importance of an intact periosteum around the fracture site, which is often damaged during surgery. And most importantly, the findings indicate that the use of recently approved anti-migraine drugs blocking either CGRP or CGRP receptor may negatively affect bone regeneration, thereby necessitating further clinical investigation.

Alt-text: Unlabelled box

## 1 Introduction

Although surgical techniques to treat bone fractures have tremendously improved in the past decades, impaired bone healing including delayed unions and non-unions following injury still represents an ongoing clinical challenge [1]. Nonunions can be observed in up to 15% of patients with fractures, even after state-of-the-art surgical and non-surgical fracture fixation, causing high socioeconomic costs and significantly lowering the quality of life of affected patients [2,3]. As treatment options remain limited, the identification of factors regulating bone repair is of high scientific and clinical interest to provide the best possible care for affected patients [4,5].

Bone healing is characterised by a cascade of well controlled complex biological processes. After injury, an acute inflammatory response marked by the release of several key cytokines and growth factors takes place and leads to the recruitment of mesenchymal stem cells to generate a primary cartilaginous callus [6]. This soft callus then undergoes revascularization and calcification, and is finally remodeled to fully restore normal bone structure. In this process, the balanced activities of bone cells, including bone-forming osteoblasts and bone-resorbing osteoclasts, are of central importance [5]. One peptide that has received increased attention in recent years due to its potential role in the regulation of bone regeneration is calcitonin gene-related peptide alpha (αCGRP). αCGRP is a neuropeptide primarily expressed in neurons of the central and peripheral nervous system through alternative splicing of the *Calca* gene transcript, which also encodes calcitonin and its precursor procalcitonin [7,8]. αCGRP was shown to regulate bone remodeling in intact bone [9], as mice lacking αCGRP display osteopenia due to a decrease in the bone formation rate [10,11]. Moreover, osteoblast-specific overexpression of CGRP resulted in an elevated bone formation [12], confirming several other *in vitro* studies that reported CGRP to promote osteoblast differentiation and function, and to enhance osteogenesis synergistically with Wnt-signaling [13], [14], [15], [16]. Of note, a close homologue to αCGRP, calcitonin gene-related peptide beta (βCGRP) is also expressed in humans and rodents. Although these peptides have a very close sequence homology, differing only in two amino acids in rodents, and are not differentiated by commercially available antibodies, these two peptides are encoded by separate genes and are expressed differently [9]. Moreover, unlike αCGPR, it is unclear whether βCGPR plays a significant role in skeletal homeostasis since mice lacking βCGPR have been shown to display only a mild and temporary decrease in bone formation [9,17]. While the role of αCGRP in bone remodeling has been intensively investigated, its role in bone regeneration following fracture remains unclear. Clinically, increased CGRP levels have been observed in patients with long-bone fractures [18,19]. Furthermore, increased in-growth of new nerve fibres containing CGRP at the fracture site have been reported in rats [20]. And finally, although inactivation of CGRP was suggested to alter M2 macrophage polarization without affecting callus maturation in ovariectomized mice [21], another study demonstrated that magnesium implants promote bone regeneration in rats through CGRP receptor-dependent, osteogenic differentiation of periosteal stem cells [22].

Aside from bone tissue, the release of CGRP from sensory nerve endings in other peripheral organs is well established and it is known to mediate biologic effects through the main CGRP receptor. The CGRP receptor is comprised of the calcitonin receptor-like receptor (CRLR) and receptor activity-modifying protein 1 (RAMP1) and its localization on the cell surface makes it an ideal drug target [23,24]. Apart from its effects in bone and other tissues, αCGRP has been primarily shown to significantly contribute to the pathogenesis of migraine, one of the most prevalent neurologic disorders estimated to affect 15% of the population worldwide [25]. During migraine attacks, CGRP levels have been reported to be increased in cranial, but not in peripheral circulation [26], [27], [28], and have been shown to cause vasodilation of cranial arterioles. Moreover, trigeminal nerve stimulation results in elevated CGRP levels in the cranial circulation [29,30], and injection of CGRP induces migraine symptoms [31].

Given the significance of CGPR peptide in migraine, the growing understanding of the CGRP signaling axis has caused excitement among health care professionals, resulting in the development of novel inhibitors of CGRP or its receptor. These drugs include gepants (e.g. olcegepant, telcagepant and ubrogepant), representing highly specific CGRP receptor antagonists, as well as monoclonal antibodies neutralizing CGRP receptor or CGRP [32]. However, while the efficacy of all these agents in migraine treatment has been demonstrated, there are concerns of liver toxicity associated with the use of gepants. Although anti-CGRP and CGRP receptor antibodies have been shown to be an excellent alternative treatment with little or no adverse effects [33], the fact that CGRP and its receptor are expressed in many different organs, including bone tissue, has raised concerns about hitherto unrecognized side effects, including a negative effect on bone fracture repair. To date, the FDA has approved one gepant (ubrogepant, targeting CGRP receptor) [34,35] and three monoclonal antibodies (erenumab, targeting CGRP receptor; galcanezumab and fremanezumab, targeting CGRP) for the preventive and acute treatment of migraine [36].

Given that novel CGRP and CGRP receptor blockers are now used clinically for migraine treatment, and the lack of direct evidence for a physiologic role of αCGRP in bone healing, our study aimed to evaluate the role of αCGRP in bone regeneration to characterize fracture healing in mice with global αCGRP inactivation. Our results demonstrate an essential role of αCGRP in bone regeneration, which suggests a potential therapeutic application of αCGRP analogues to boost fracture healing and warrants further clinical studies to evaluate the effects of CGRP and CGRP receptor inhibitors on bone regeneration.

## 2 Materials and methods

### 2.1 Animals

For all experiments in this study, a total of 90 12-week-old female mice, which included 59 wild type (WT) and 31 mice with global inactivation of αCGRP expression (αCGRP^−/−^ mice) were used (**Suppl. Tab. 1**). The generation and genotyping of αCGRP^−/−^mice was described previously [37]. A separate strain of WT mice was employed which was generated out of a heterozygote αCGRP^+/−^ mating. All mice were backcrossed to a pure C57Bl/6 J background at least 7 times. αCGRP^−/−^ and WT controls were kept at a 12 h light /12 h dark cycle and fed a standard diet and water ad libitum. Unless stated otherwise, 6 mice per group were used for in vivo analyses (*n* = 6). Missing data points are explained by sample artefacts after processing not allowing adequate histomorphometric quantifications or by insufficient sample quality in case of RNA extraction. All animal experiments were approved by the local legal representative animal rights protection authorities (G0277/16) and performed adherent to the policies and principles established by the animal Welfare Act (Federal Law Gazett I, p.1094) and the national institutes of health guide for care and use of laboratory animals.

### 2.2 Surgical procedure

Bone injury was induced through a femoral osteotomy stabilized with an external fixator of the left limb using a standardized model as described previously (**Suppl. Fig. 1a**) [38]. For the mid-diaphyseal approach a lateral longitudinal skin incision (2 cm length) along an imaginary line from the knee to the hip joint was performed. The femoral bone was exposed by dissection of the fascia lata and by blunt preparation of the Musc. vastus lateralis and the Musc. biceps femoris sparing the sciatic nerve. The first pin hole was drilled with a fine hand-drill (diameter: 0.45 mm) just proximal to the distal metaphysis of the femur, perpendicular to the longitudinal femoral axis and cortical surface. Thereafter, serial drilling for pin placement through the connectors of the external fixator (RISystem) was conducted, resulting in a fixation of the external fixator construct strictly parallel to the femur. Following rigid fixation, a 0.70 mm osteotomy was performed between both middle pins using a Gigli wire saw (RISystem). Wounds were closed with Ethilon 5–0 suture.

Fracture healing, including serum sampling and tissue harvesting, was evaluated after 7, 14, and 21 days post-surgery (*n* = 6 per genotype and time point), representing the acute inflammation, the soft callus, and the remodeling stage of bone regeneration, respectively. Moreover, for gene expression studies, callus and organ tissues were sampled also at day 3 and analysed as indicated. Euthanasia was performed in CO_2_. The intact femur and other tissues of untreated mice with the same sex and age were used as controls. The femur including the external fixator was separated from knee and hip joint, and the surrounding tissue was removed carefully. The femur including the hardware were secured in a sliced plastic pipette, followed by removal of the external fixator for µCT analysis. Thereafter, bones were cyro-embedded for histologic investigation and immunologic protein detection. For genome-wide expression analysis, an extra set of 3 mice per genotype was used (*n* = 3).

### 2.3 Gene expression analysis

Bones were carefully dissected from soft tissue and the callus tissue between the two middle pins was extracted using a scalpel (please compare **Suppl. Fig. 1a**). Callus and other tissues were snap-frozen and homogenized in TRizol using a UltraTurrax (Sigma Aldrich), followed by the isolation of total RNA using RNasy mini Kit (Qiagen). Complementary DNA (cDNA) was synthesized using RevertAid First Strand cDNA Synthesis Kit (ThermoFisher). Quantitative real-time PCR (qRT-PCR) was carried out using Power SYBR Green PCR Master Mix (Sigma Aldrich) or TaqMan Assay-on-Demand primer sets supplied by Applied Biosystems (*Ramp1, Runx2, Col1a1, Alpl, Sp7, Bglap, Adipoq, MMP13, Mrc1, Il1b*).

Gene expression was calculated as virtual copy number per housekeeper gene *Glyceraldehyde-3-phosphate dehydrogenase* (*Gapdh*) or fold expression as indicated by the ΔΔCT method. For SYBR Green assays, the following primer sequences were applied: *Gapdh* forward ACTGAGCAAGAGAGGCCCTA, *Gapdh* reverse TATGGGGGTCTGGGATGGAA, *Calca* forward AAGGGAGCACGTGTTATGGT, *Calca* reverse TCCATTCTGAATTGAGGGTGGG, *Crlr* forward GGGGACAGTTACATGAGTCCA, *Crlr* reverse GGGCTGAGTCACTCCTCTCA, *Cfd* forward GCATGGATGGAGTGACGGA, *Cfd* reverse ACCATCGCTTGTAGGGTTCAG, *Ccl7* forward CAACCTAGGAGCCAAGAAGCA, *Ccl7* reverse AGCTCCTATCCCTTAGGACCG, *Scd1* forward TGTTATAGACGGCAGTTGGCA, *Scd1* rev ACACCACCTCACTGGAGCTA.

Genome wide expression analysis was performed using the Clariom™ D array kit (Thermo Fisher Scientific, Inc.) according to the manufacturer's instructions. In brief, RNA quality and integrity were assessed by spectrophotometry (Nanodrop Technology, Inc.) and Tapestation 2200 (Agilent Technolgies, Inc.). The RIN range of the samples used for further assessment was 7.8–8.2. 100 ng of total RNA from whole calli was used as input for cRNA synthesis and subsequently 15 µg of cRNA were used for cDNA synthesis. The cDNA was fragmented and labelled prior to micro array hybridization at 45 °C for 16 h. After washing and staining of the micro arrays in the Affymetrix Fluidics Station 450 (Thermo Fisher Scientific, Inc.), they were scanned with a GeneChip Scanner 3000 7 G (Thermo Fisher Scientific, Inc.). Data analysis was performed in the Transcriptome Analysis Console v. 4.0.1.36 (Thermo Fisher Scientific, Inc.) using default settings. Average fold-change values were calculated using Tukey's bi-weight average algorithm. For all displayed genes, FDR-adjusted *p* > 0.1. Full datasets are available in the GEO repository GSE148720.

### 2.4 Serum analysis

ELISA for serum CGRP levels (not differentiating between α and βCGRP) was conducted using a CGPR (rat, mouse) EIA-Kit (K-015–09, Phoenix Pharmaceuticals) following kit instructions.

### 2.5 μCT analysis

To evaluate the formation of newly formed bone in the fracture gap, micro-computer tomography (μCT) analyses were performed on bone samples secured in plastic tubes for stabilization of the callus at an isotropic voxel size of 10.05 µm, 80 kV and 124µA (Skyscan 1172F). The scan axis coincided with the diaphyseal axis of the femora. All analyses were performed on a volume of interests (VOI) compromising 100 slices containing the callus and cortices at a global threshold of 40 – 100 at 16 bit stacks using CTan v 1.18. 8 (please also refer to **Suppl. Fig. 1b**) [39]. Data is reported according to the guidelines for tissue imaging by the American Society of Bone and Mineral Research [40].

### 2.6 Histomorphometric analysis

Histological analysis was performed on harvested bones at 7, 14 and 21 days post-surgery. After bones were used for the µCT analysis they were subjected to the histomorphometric investigation. In this regard, skeletal tissue was fixed overnight in 4% PFA, followed by incubation in an accenting sugar gradient (10%, 20% and 30% each for 24 h). The dehydrated bones were placed longitudinal with the fixator holes pointing upwards in a mold, immerged with SCEM embedding medium (Section Lab Co Ltd.) and frozen over cooled hexane (Carl Roth GmbH&CoKG). After hardening, the bones were cut longitudinally in transversal plane in 5 µm sections using a cryotome (Leica CM3050S, Leica Microsystems). The sections of the same area (transversal visible bone marrow with 4 cortice) were mounted on microscope slides using cryofilm (Cryofilm type II C, Section Lab Co Ltd.), and Movat Pentachrome staining was performed for histomorphometry analysis. For this, sections were stained in alcian blue (8GS, Chroma), Weigert's haematoxylin (Merck), Brilliant Crocein/Acid Fuchsin (Brilliant Crocein R, Chroma, and Acid Fuchsin, Merck), 5% Phosphotungstic acid PTA (Chroma), and Saffron du Gâtinais (Chroma). The stained sections were mounted in Vitro-Clud (Langenbrinck). For histomorphometry, mosaic images were taken using the Axioscop 40 (Zeiss) and Axiovsion Rel.4.8 software. Static and cellular histomorphometry were performed in the region of interest (ROI) of the fracture gap (please see **Suppl. Fig. 1c**) of one section per animal and time point (total 18 sections per genotype) using ImageJ software. Callus bridging was evaluated with the following scoring: *A* = complete bridging (all four cortices bridged by callus), *B* = partial bridging (two to three cortices bridged by callus), *C* = incomplete bridging (callus present, but no bridging visible), and *D* = non-union (rounded cortices, minimal presence of callus). Scoring was performed independently by two blinded reviewers. The mean value of the observation is depicted as stacked bar charts [41].

### 2.7 Immunohistochemistry

To localize osteocalcin-expressing cells, chromogen osteocalcin staining was performed. Specifically, frozen sections were fixed in 4% PFA, washed in PBS and permeabilized in 3% H_2_0_2_, followed by washing with PBS and blocking in 1% BSA/PBS with 5% goat serum. The sections were then incubated with primary anti-osteocalcin antibody (1:4000, cat# ALX-210–333, RRID:AB_2052106). At the next day, sections were washed in PBS, incubated with the biotinylated *sec*. antibody (anti-rabbit,1:200,  Cat# BA-1000, RRID:AB_2313606), washed in PBS and incubated with avidin-peroxidase conjugate (ABC-Kit, PK6100, Vetor). Detection was performed through a commercial kit (Kit SK 4100, Vector) and a brief counterstain with Meyers haematoxylin. For TRAP activity staining, sections were fixed with 4% PFA and titrated to pH 5 using a buffer containing sodium acetate (Merck) and sodium tartrate-dehydrate (Merck). After washing with distilled water, sections were stained in the presence of Naphtol AS-MIX- Phosphate, Fast Red Violet LB Salt, N,N-Dimethylromaid and Triton X (Sigma Aldrich). A counterstain was carried out with Mayer's Hemalaun solution (Merck). Osteoclasts were identified as TRAP-positive cells with ≥ 3 nuclei and adherent to the bone surface. Osteoblasts were identified as mononuclear cells adherent to the bone surface with typical cuboidal morphology. Static and cellular histomorphometric parameters were assessed according to the guidelines of the American Society for Bone and Mineral Research using ImageJ software [42].

### 2.8 Fluorescent immunohistochemical staining

For localization of CGRP (not differentiating between alpha- and beta-CGRP), Endomucin (Endm) and CD31 within the fracture gap, sections were permeabilised with 0.25% Triton/PBS for 10 min. After permeabilization, sections were washed with PBS and blocked in 3%BSA/5% Donkey Serum/PBS. Following primary antibody incubation with anti-CGRP (1:100, cat# ab47027, RRID:AB_1141573), anti-CD31 (1:100, cat# AF 3628, RRID:AB_2161028), and anti-Endm (1:100, cat# sc-65495. RRID:AB_2100037) overnight, sections were washed in PBS and incubated with secondary antibody (1:400, anti-rabbit 647,  Cat# A32728, RRID:AB_2633277; 1:400, anti-goat 546, Cat# A-11058, RRID:AB_2534105; 1:400, anti-rat 488, Cat# A-21208, RRID:AB_2535794) and mounted in Fluromount-G with DAPI (Thermo Fisher Scientific, Inc.). For localization of the CGRP receptor (CRLR/RAMP1 complex) within the fracture, gap serial sections were used. For staining of CRLR, sections were permeabilized with PBS/0.5% Triton for 10 min and washed with PBS/0.25%Triton prior to blocking in 3%BSA/5% donkey serum with 0.1% Triton. After blocking, the sections were incubated overnight with primary anti-CRLR antibody (rabbit) (1:200, bs-1860R-TR, Bioss Antibodies). Following subsequent washing, application of secondary antibody (1:400, anti-rabbit-Cy3, 711-165-152, Dianova; RRID:AB 2307443), and nucleus staining was performed as described above. For staining of RAMP1, sections were washed with PBS, blocked in 3%BSA/5% donkey serum and incubated in RAMP1 antibody (rabbit) (1:750, ab156575, Abcam; RRID:AB_2801501) over night. Following incubation, the sections were washed with PBS/0.1% Tween20 with subsequent incubation of the secondary antibody (1:400, anti-rabbit-Cy3, 711-165-152, Dianova; RRID:AB 2307443) and nucleus stained as outlined above. Images were acquired using Leica DM RB microscope (Leica Microsystems) and Axiovision Rel. 4.8 software (Zeiss) as well as LEICA SP5 confocal microscope equipped with a Mai Tai HP multiphoton laser (Spectra Physics).

### 2.9 Cell culture

Osteoclast precursor cells were isolated from flushed bone marrow of 10 to 14- week-old female mice and differentiated for 2 days in α-MEM containing 10 nM 1,25(OH)_2_ Vitamin D_3_ at a density of 1 × 10^6^ cells/ml in 24-well plates. The culture medium was supplemented with M-CSF (20 ng/ml; PeproTech) and RANKL (40 ng/ml; PeproTech) and cells were cultured for additional 4 days. The cultured medium was changed every other day, which included the removal of non-adherent cells to allow terminal osteoclast differentiation. Osteoclast formation was quantified by TRAP activity staining as described previously [43]. In brief, after removal of the medium and two washing steps with PBS, cells were fixed with cold methanol for 5 min. After washing and drying, cells were stained with Naphthol AS-MX-Phosphate (Sigma) for 30 min and the number of TRAP-positive multinuclear cells per well (*n* > 3 nuclei/cell) determined.

Bone marrow-derived osteoblasts were generated by differentiation of flushed bone marrow cells in culture (1 × 10^6^ cells/ml in 24-well plates). The isolated cells were cultured in α-MEM supplemented with 25 μg/ml ascorbic acid and 5 mM β-glycerophosphate. The culture medium was changed every other day, which included the removal of non-adherent cells. The cultured cells were either treated with or without CGRP (10^−7^ M; Bachem) and/or olcegepant (1μg/ml; Tocris) as indicated. At the end of the culture, cells were fixated in 90% ethanol for 1 h and then washed twice in distilled water. Cells were then incubated with 40 mM alizarin red staining solution (pH 4.2) for 10 min at room temperature. To quantify alizarin red incorporation, cells were washed with PBS and fixed in 90% ethanol for 1 h. After washing twice with distilled water, cells were stained with alizarin red S solution (40 mM, pH 4.2) for 10 min. Following additional washing steps with distilled water, cell-bound alizarin red was dissolved in 10% acetic acid and the samples incubated for 30 min at room temperature and 10 min at 85  °C. After centrifuging, the supernatant was neutralized with 10% ammonium hydroxide solution and absorbance measured at 405 nm. For short-term treatment, osteoblasts were serum-starved overnight at the indicated differentiation stages and stimulated with CGRP (10^−7^ M) with or without olcegepant (1μg/ml) for 6 h. For each experiment, two technical replicates were performed. Biological replicates were based on pooled cells derived from 3 to 4 mice.

### 2.10 Statistical analysis

For two-group comparisons, data were analysed by two-tailed Student's *t*-test using Graphpad Prism software. Due to loss or destruction of samples, the number of available samples for analyses varied and is indicated with individual data points for each experiment. In case of multiple comparisons, one-way or two-Anova followed by Tukey post-hoc analysis was applied as indicated. If not stated otherwise, all data are box plots with median value and maximum and minimum whiskers. *p* < 0.05 was considered statistically significant.

## 3 Results

### 3.1 CGRP and its receptor CRLR are expressed in regenerating bone

**Fig. 1:**
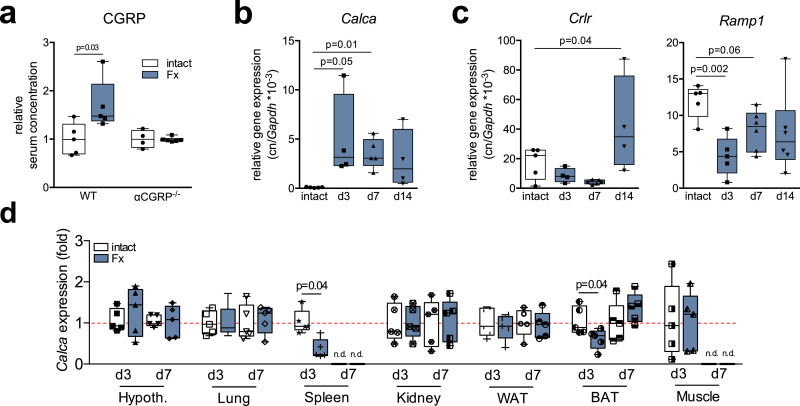
CGRP and CGRP receptor are expressed in the fracture callus on mRNA level. (**a**) Relative serum CGRP levels in WT and αCGRP-deficient mice with a femoral osteotomy stabilized with an external fixator 14 days post-injury. *n* = 4–6 mice per group (unpaired student's *t*-test). (**b, c**) Gene expression (virtual copy numbers per *Gapdh*) of the indicated genes in the intact femoral shaft of control animals (intact) and in the fracture callus at the respective time points during bone regeneration (d3, d7, d14). *Calca* = encoding αCGRP; *Crlr* = calcitonin receptor-like receptor; *Ramp1* = receptor activity-modifying protein 1. *n* = 4–6 mice per group (one-way Anova followed by Tukey post-hoc test). (**d**) *Calca* expression (fold) in the indicated tissues 3 and 7 days following Fx. Hypoth. = hypothalamus; WAT = white adipose tissue; BAT = brown adipose tissue; n.d. = not detectable. The dotted red line indicates average expression of respective, untreated control tissues. *n* = 3–5 mice per group (unpaired student's *t*-test). For (**a**-**d**), box plots represent median with minimum and maximum whiskers. Controls are untreated mice of the same sex and age.

In order to assess the role of αCGRP in bone regeneration, we first employed ELISA to measure CGRP levels in the serum of WT mice with a femoral fracture stabilized with an external fixator (Fx mice). Since the primary antibody used is presumed to recognize both α and βCGRP, the term CGRP will be used hereafter for the sake of clarity with regards to protein detection. Confirming clinical observations in patients with a proximal fracture of the femur [18,19], Fx mice showed increased CGRP levels 14 days following surgery (Fig. 1**a**). In order to rule out an involvement of βCGRP, we employed mice with global inactivation of αCGRP but intact βCGRP expression. These mice carry a stop codon immediately upstream of the coding region of the αCGRP alternative splice transcript in exon 5 [11,37]. This renders the expression of the other *Calca*-encoded peptides (calcitonin and its precursor procalcitonin) intact and exclusively inactivates the expression of αCGRP. In contrast to WT controls, CGRP levels were not elevated in αCGRP-deficient mice at day 14 following the osteotomy. This suggests that the increased CGRP concentrations in WT mice is indeed due to an increase in the levels of αCGRP and not βCGRP. Analysis of gene expression in the fracture callus revealed an induction of *Calca*, encoding αCGRP, at day 3 following surgery compared to intact bone, which slightly declined thereafter during the course of bone regeneration (Fig. 1**b**). In contrast, *Calcrl* mRNA, encoding CRLR, was expressed at lower levels at day 3 and 7, however it was expressed at significantly higher levels at day 14 following Fx compared to intact bone (Fig. 1**c**). Similarly, the expression of *Ramp1* in the fracture callus was lower at day 3 compared to those in intact bone and gradually increased during bone healing. To rule out a different source for the increased CGRP levels observed in the serum following Fx, we also monitored *Calca* expression in non-skeletal tissues during bone generation in WT mice. While Fx resulted in decreased *Calca* expression in spleen and brown adipose tissue at day 3, no induction was observed in any of the tissues studied at day 3 or day 7, indicating that increased *Calca* expression following Fx is limited to the callus (Fig. 1**d**).

**Fig. 2:**
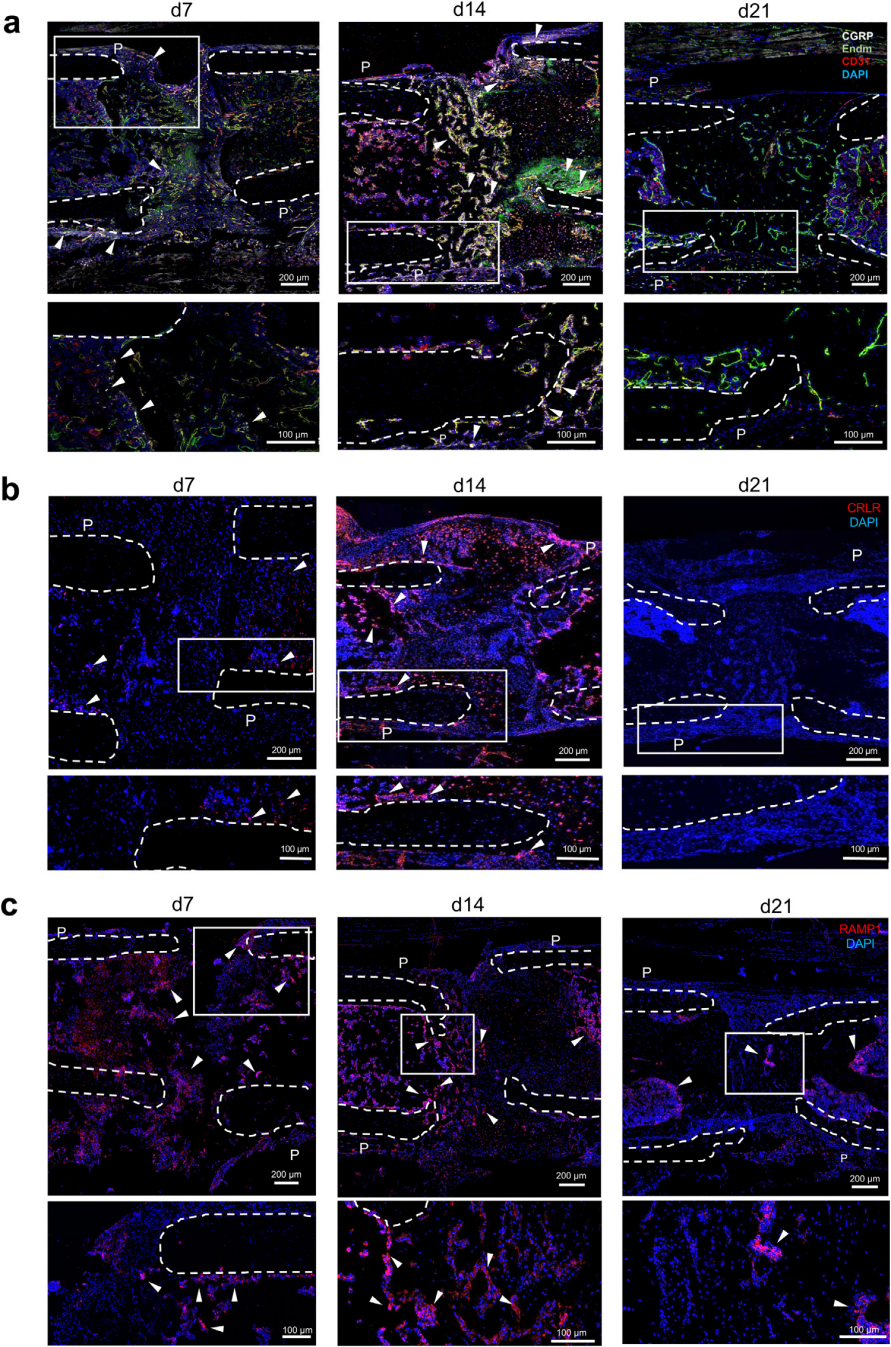
CGRP and CGRP receptor are expressed in the fracture callus on protein level. (**a**) Representative immunofluorescent stainings (merged) of WT callus sections 7, 14, and 21 days after surgery using a CGRP-, endomucin- (Edm) and CD31-specific antibody. (**b, c**) Representative immunofluorescent stainings of WT callus sections 7, 14 and 21 days after surgery using a CRLR or RAMP1-specific antibody as indicated. *P* = periosteum. White boxes of the upper row show the magnified area illustrated below. Arrows indicate CGRP-, CRLR- orRAMP1-positive structures, respectively, and dotted white line show the fracture ends.

We next studied the expression of CGRP and its receptor on protein level using immunostaining from sections derived from Fx mice 7, 14 and 21 days post-surgery. CGRP immunoreactivity was low in intact femur (**Suppl. Fig. 2**) but high in healing fracture on day 7 and 14. However, at day 21, CGRP immunoreactivity was almost undetectable. Confocal microscopy showed that CGRP was predominantly expressed in the periosteum in close vicinity of the fracture site and within the fracture callus co-localized with invading blood vessels that are known to be richly innervated by nerve fibres (merged images Fig. 2**a;** individual channels see **Suppl. Fig. 3**). In the case of the CRLR component of the CGRP receptor, immunoreactivity was low on day 7 around the fracture site, but stronger signals were detected at day 14 in the callus and the periosteum around the fracture (merged images Fig. 2**b;** individual channels please see **Suppl. Fig. 4a**). This was in line with the results obtained through qRT-PCR. Similarly, no immunoreactivity against CRLR was detected in the fracture callus after 21 days. In the case of the RAMP1 component of the CGRP receptor, immunoreactivity was moderate on day 7 around the fracture site, but strong signals were detected on day 14 in the callus and the periosteum around the fracture (merged images Fig. 2**c;** individual channels please see **Suppl. Fig. 4b**). Collectively, these results demonstrate that αCGRP and its receptor are expressed in callus tissue, particularly during the early and intermediate regenerative stages of bone repair.

### 3.2 Bone regeneration is profoundly impaired in αCGRP-deficient mice

**Fig. 3:**
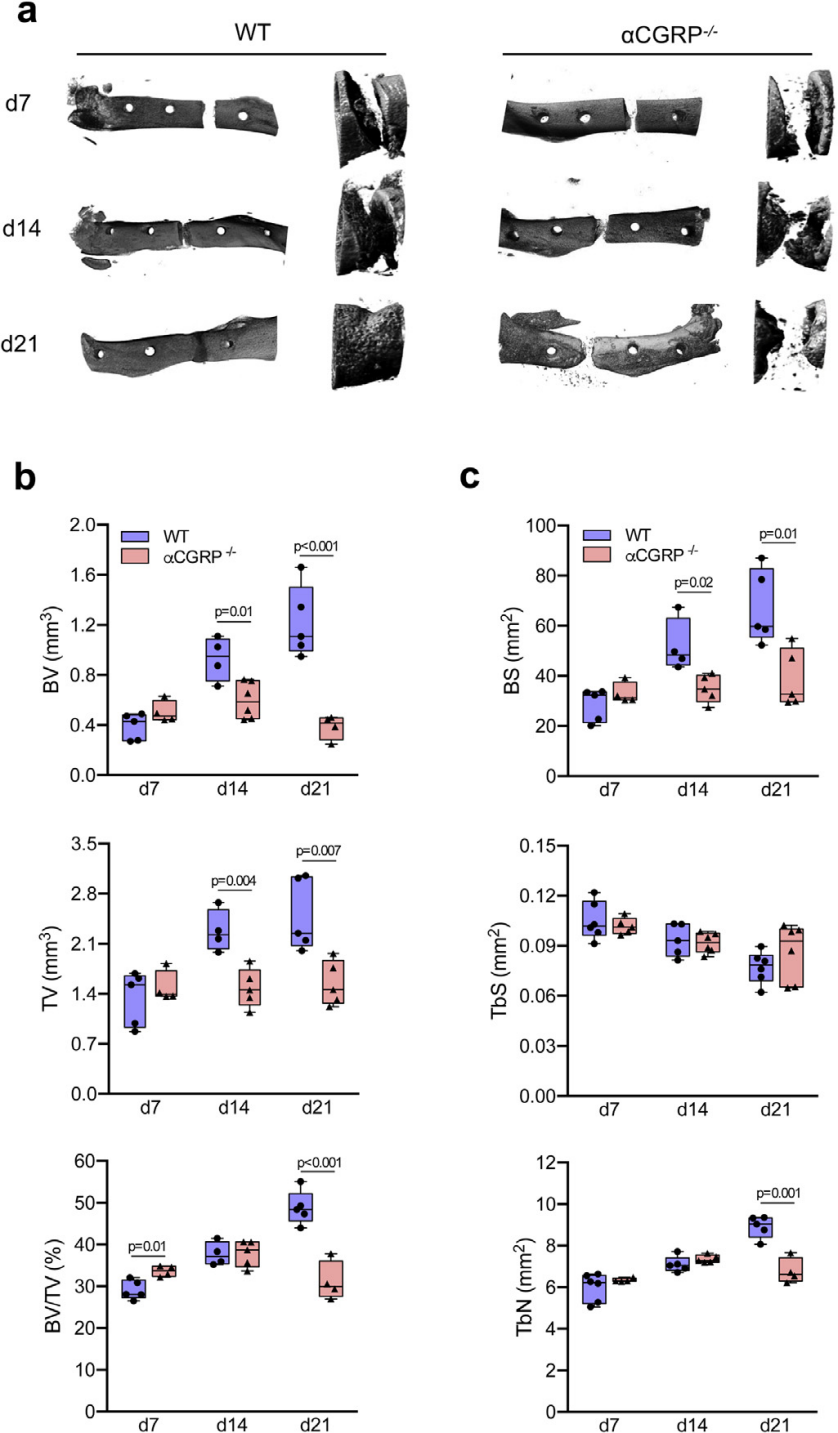
Deficiency in αCGRP results in the failure of bone regeneration. (**a**) Representative µCT images (left column= longitudinal overview; right column = longitudinal magnification) of the callus region in the femur of WT and αCGRP-deficient mice at the indicated time points. (**b**) Quantitative analysis of µCT images in mice of both genotypes at the same time points. BV = total callus bone volume, TV = total tissue volume, BV/TV = bone volume vs. tissue volume. (**c**) Quantitative analysis of µCT images in the same samples. BS = bone surface, TbS = trabecular surface, TbN = trabecular numbers. For (**b**) and (**c**), the different timepoints were evaluated independently and compared by unpaired student's *t*-test. *n* = 4–6 as indicated per group and time point. Box plots represent median with minimum and maximum whiskers.

Mice with global inactivation of αCGRP expression were used to study the role of αCGRP in fracture repair. For the *in vivo*-assessment of bone regeneration, αCGRP-deficient and WT mice were subjected to Fx and then euthanized after 7, 14, and 21 days for morphological assessment. Radiological analysis of bone healing using μCT revealed a reduced regenerative capacity and delayed healing process in αCGRP-deficient mice (Fig. 3**a**). Although a small but significant increase in bone volume vs. tissue volume (BV/TV) was detected at day 7 post injury, αCGRP-deficient mice showed an insufficient callus formation after 14 and 21 postoperative days. This was evident in the reduction in total callus bone volume (BV) and tissue volume (TV), that was coupled with reduced BV/TV on day 21 (Fig. 3**b**). In line with this, a reduced bone surface (BS) and a tendency towards a lower trabecular surface (TS) with a reduction in trabecular numbers (TbN) was detected on day 21 in the fracture callus of αCGRP-deficient mice (Fig. 3**c**).

**Fig. 4:**
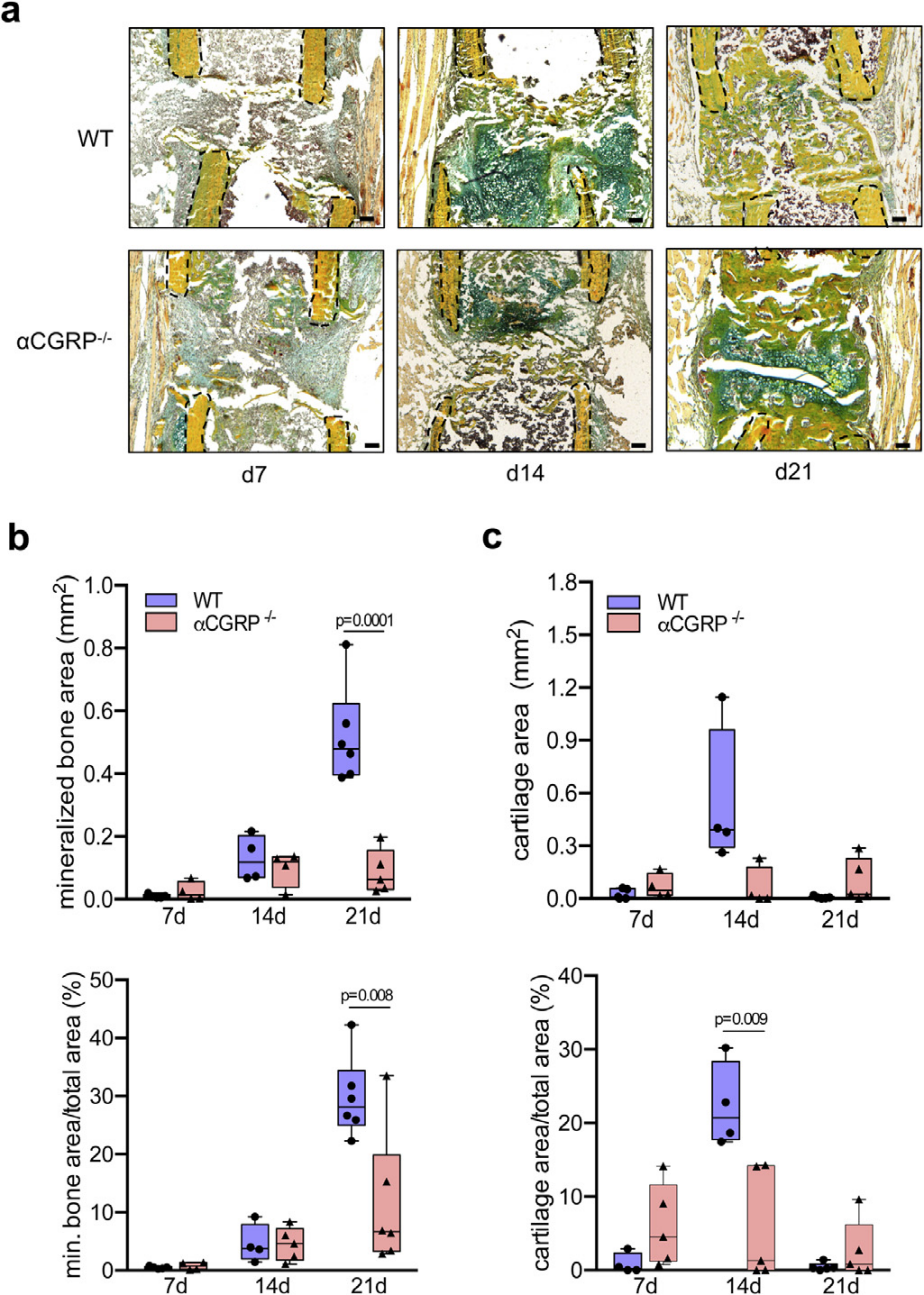
Impaired callus formation in αCGRP-deficient mice. (**a**) Representative callus sections (Movat Pentachrome staining) of WT and αCGRP-deficient mice at the indicated time points (yellow = mineralized bone; green = cartilage; red = muscle). Black dotted line indicates fractured bone cortices within the developing callus. (**b, c**) Histomorphometric quantification of static callus parameters in the same mice. Scale bars = 200 μm. *n* = 4–6 as indicated per group and time point. Box plots represent median with minimum and maximum whiskers. For (**b**) and (**c**), the different timepoints were evaluated independently and compared by unpaired student's *t*-test.

This result was collaborated by the findings from histological analysis of non-decalcified callus sections, which showed impaired bone regeneration in αCGRP-deficient mice compared to control mice at day 14 and 21 post injury (Fig. 4**a**). The histomorphometric quantification revealed a reduced amount of mineralized bone (mineralized bone area) and a lower percentage of mineralized bone in the total callus area (mineralized bone area/total area) in mutant mice (Fig. 4**b**). In the case of cartilage, a trend towards lower cartilage area and a significantly reduced cartilage area per total area was observed in the fracture gap of αCGRP-deficient mice at day 14 post-surgery (Fig. 4**c**). However, a trend towards an increase in these parameters was detected on day 21, suggesting impaired cartilaginous callus remodeling in αCGRP-deficient mice.

### 3.3 Insufficient callus bridging in αCGRP-deficient mice

**Fig. 5:**
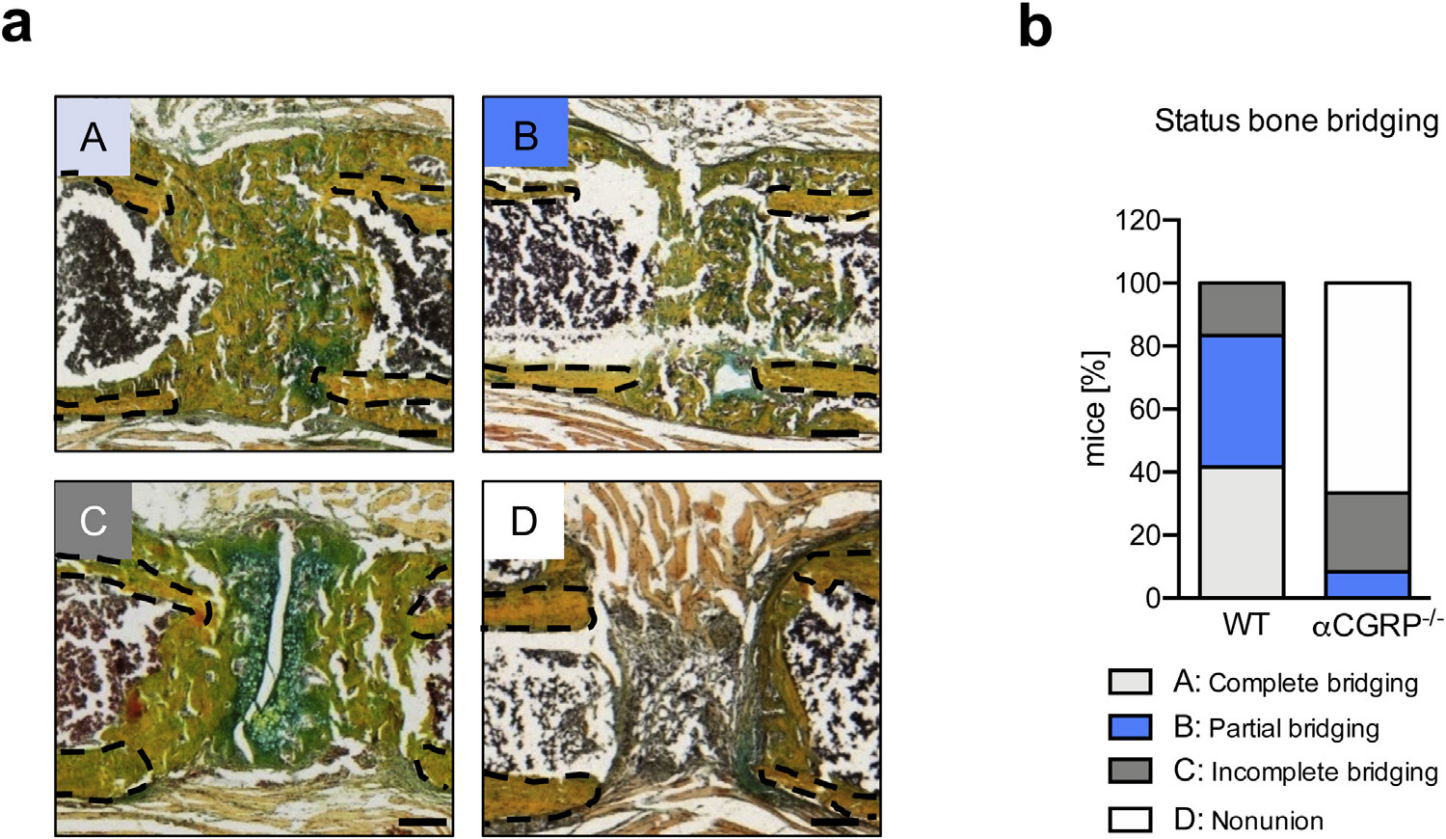
High rate of fracture non-union in αCGRP-deficient mice. (**a**) Exemplary callus images (Movat Pentachrome staining) representing the different outcomes of callus union at day 21 following Fx: *A* = complete bridging (all four cortices bridged by callus), *B* = partial bridging (two to three cortices bridged by callus), *C* = incomplete bridging (callus present, but no bridging visible), and *D* = non-union (rounded cortices, minimal presence of callus). Black dotted line indicates fractured bone cortices within the developing callus. (**b**) Semiquantitative evaluation of callus bridging in WT and αCGRP-deficient mice at the indicated time points. *n* = 6 mice per group. Scale bars = 200 μm.

In the assessment of the rate of non-union in mice using a semi-quantitative scoring of osseous callus bridging on day 21 following Fx (Fig. 5**a**), most WT control mice displayed complete (41.66%) or partial (41.66%) bridging of the fracture ends, and a small percentage (16.66%) displayed delayed union (Fig. 5**b**). In contrast, bone regeneration in αCGRP-deficient mice was characterized by a high number of delayed unions (25%) and non-unions (66.66%), with only a small percentage of animals reaching partial bridging (8.3%) and none achieving complete union. Collectively, these findings indicated that callus formation is severely impaired in αCGRP-deficient mice, resulting in a high rate of fracture non-union, possibly due to disturbed remodeling processes in the regenerating bone.

### 3.4 αCGRP-deficient mice display impaired cellular callus formation

**Fig. 6:**
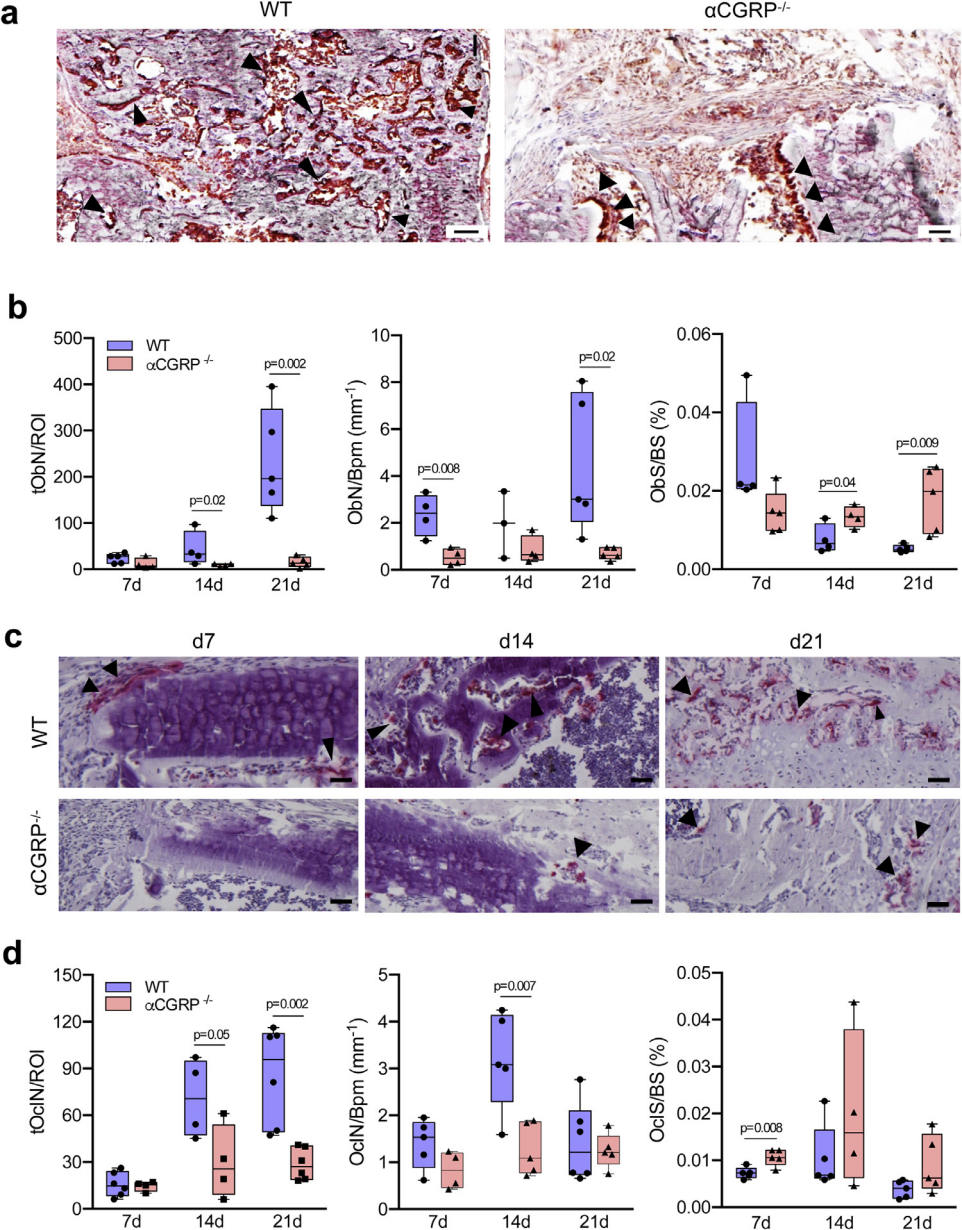
αCGRP-deficient mice display profound alterations in callus bone cell distribution. (**a**) Representative callus images demonstrating osteocalcin-positive (brown), bone-forming osteoblasts (black arrows) in WT and αCGRP-deficient mice at d21 following surgery. Scale bars = 100 μm. (**b**) Histomorphometric quantification of osteoblast parameters in the callus of the same mice at the indicated time points. tObN/ROI = total osteoblast numbers in the callus area; ObN/Bpm = osteoblast numbers per bone perimeter; ObS/BS = osteoblast surface per bone surface. (**c**) Representative callus images (TRAP activity staining) demonstrating tissue-resorbing osteoclasts (red staining, arrows) in WT and αCGRP-deficient mice at the indicated time points. Scale bars = 200 μm. (**d**) Histomorphometric quantification of osteoclast parameters in the callus of the same mice. tOcN/ROI = total osteoclast numbers in the callus area; OcN/Bpm = osteoclast numbers per bone perimeter; OcS/BS = osteoclast surface per bone surface. For (**b**) and (**d**), *n* = 4–6 as indicated per group and time point. The different timepoints were evaluated independently and compared by unpaired student's *t*-test. Box plots represent median with minimum and maximum whiskers.

As these findings pointed towards an insufficient bone cell function in the callus of mutant animals, we next assessed cellular osteoblast and osteoclast parameters in the fracture gap using osteocalcin and TRAP activity staining, respectively. Histologic sections indicated a striking decrease in the amount of osteocalcin-positive, bone forming osteoblasts after 21 days following surgery in αCGRP-deficient mice (Fig. 6**a**; for negative control please see **Suppl. Fig. 5**). The fact that quantitative histomorphometry revealed a significant reduction in the total number of osteoblast in the callus area (tObN/ROI) at day 14 and 21 post injury points towards a dysfunctional formation of new bone during fracture healing in mutant animals (Fig. 6**b**). This observation was supported by reduced osteoblast numbers per bone perimeter (ObN/Bpm) at day 7 and 21. Osteoblast surface (ObS/BS) showed a trend towards lower values in αCGRP-deficient mice compared to WT controls at day 7, although these significantly increased at day 14 and 21 post injury.

The evaluation of cellular bone resorption parameters by analysing TRAP-positive, multinucleated cells (*n* > 3 nuclei per cell) adherent to the bone surface showed profound alteration in the number of osteoclasts within the fracture site associated with αCGRP-deficiency (Fig. 6**c**). Quantitative histomorphometry confirmed a significant reduction in the total number of osteoclasts within the fracture site at day 14 and 21, which was accompanied by a lower number of osteoclasts per bone perimeter (OcN/BpM) on day 14 (Fig. 6**d**). Moreover, an increase in osteoclast surface (OcS/BS) detected on day 7 following osteotomy suggests possible compensatory mechanisms and an overall insufficient callus organization in αCGRP-deficient mice.

### 3.5 CGRP-CRLR signaling is required for the expression of pro-osteogenic mediators associated with the PPARγ pathway in the fracture callus

**Fig. 7:**
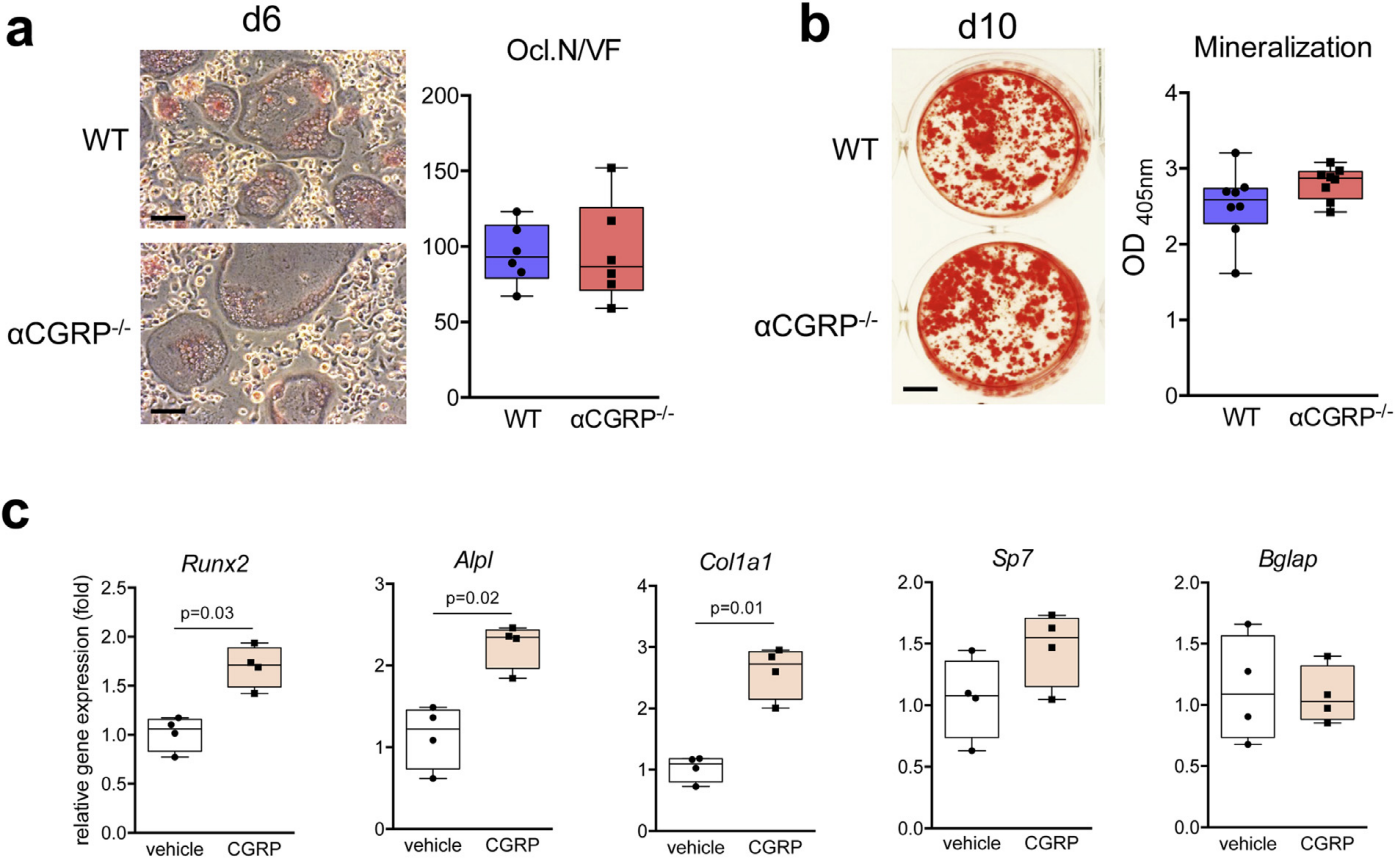
Osteogenesis and osteoclastogenesis is not affected in bone-marrow cells derived from αCGRP-deficient mice. (**a**) TRAP activity staining of WT and αCGRP-deficient, bone marrow-derived osteoclasts differentiated in the presence of M-CSF and RANKL (staining performed at day 6 of differentiation). Scale bars = 50 μm. The quantification of osteoclast numbers per viewing field is depicted on the right (Ocl.N./VF). *n* = 6 independent cultures per group. (**b**) Alizarin red staining of bone marrow-derived osteoblasts from the same mice differentiated in the presence of ascorbic acid and β-glycerophosphate at day 10 of osteogenic differentiation. Scale bar = 4 mm. The quantification of extracellular matrix mineralization is depicted on the right. *n* = 8 independent cultures per group. (**c**) qRT-PCR expression analysis (fold*)* for the indicated genes in bone marrow-derived osteoblasts at day 2 of osteogenic differentiation with ascorbic acid and β-glycerophosphate, stimulated with CGRP (10^−7^ M) for 6 h. *n* = 4 independent cultures per group (unpaired student's *t*-test).

Although CGRP has previously been reported to stimulate the osteogenic differentiation of mesenchymal stem cells *in vitro* [13], [14], [15], [16] and to promote magnesium-induced bone formation *in vivo* [*22*], its role in orchestrating callus formation remained largely unknown. In the study to rule out a cell-autonomous defect in αCGRP-deficient bone cells, isolated bone marrow cells from αCGRP-deficient and control WT mice differentiated *in vitro* into osteoclasts and osteoblast showed no difference in osteoclast formation (Fig. 7**a**) or osteoblast matrix mineralization (Fig. 7**b**), respectively. This demonstrates intact osteogenic differentiation and osteoclastogenesis in mutant cells. However, short-term stimulation of bone marrow cells undergoing early osteogenic differentiation with recombinant αCGRP resulted in an enhanced mRNA expression of the key osteoblast markers *Runx2, Col1a1*, and *Alpl* (but not *Sp7* or *Bglap*), which are involved in osteoblast differentiation, extracellular matrix formation and mineralization, respectively (Fig. 7**c**). These observations suggested, that the profound phenotype of αCGRP-deficient mice is not inherently caused by a cell-autonomous defect in their bone marrow cells, but rather by the absence of αCGRP and its function as a locally secreted ligand orchestrating callus formation.

**Fig. 8:**
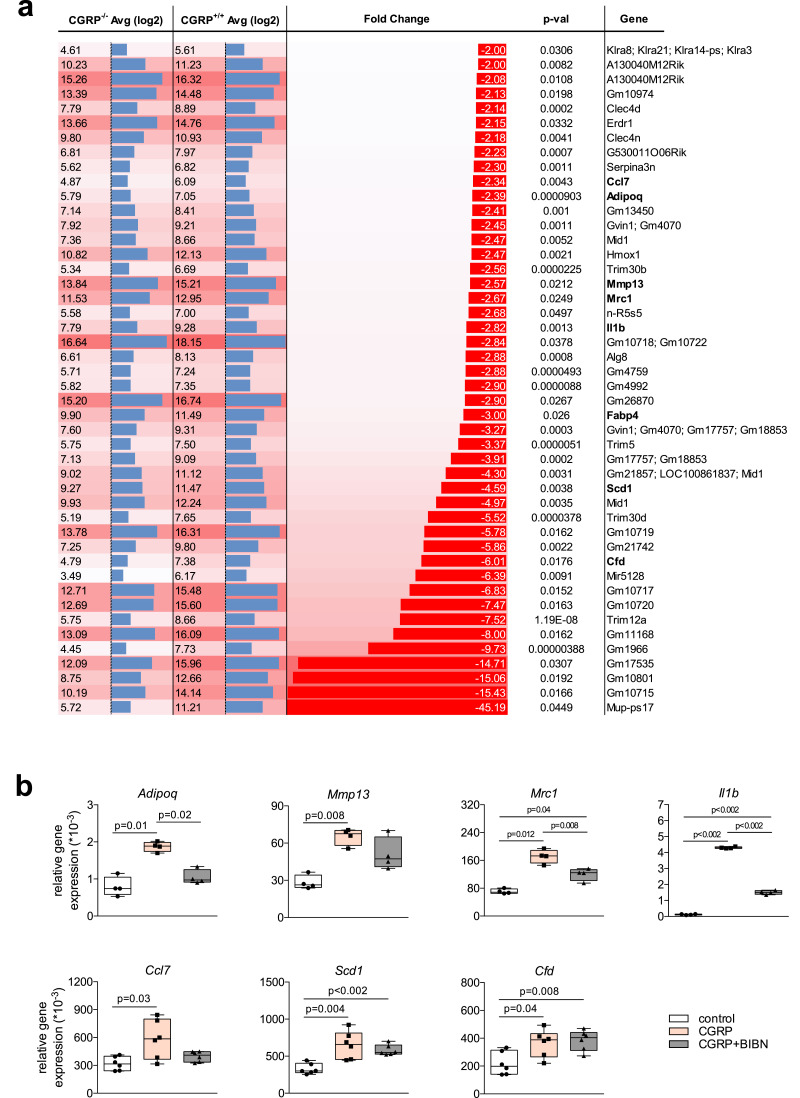
Decreased expression of genes associated with bone formation, remodeling, and PPARγ signaling in the callus of αCGRP-deficient mice. (**a**) Average (Avg log2) and relative (fold) expression of genes with significant reduction in day 7 callus tissue from 12-week-old female mice using genome-wide expression analysis (αCGRP-deficient mice vs. WT mice; *n* = 3 per genotype). (**b**) qRT-PCR expression analysis (virtual copy numbers per *Gapdh)* for the indicated genes in bone marrow-derived osteoblasts at day 2 of osteogenic differentiation with ascorbic acid and β-glycerophosphate, stimulated with αCGRP (10^−7^ M) and olcegepant (1μg/ml; BIBN) for 6 h as indicated. *n* = 4–6 independent cultures per group (two-way Anova followed by Tukey post-hoc test).

A close scrutiny of the RNA isolated from the callus of control WT and αCGRP-deficient mice (*n* = 3 per genotype) on day 7 of fracture healing to study genome-wide gene expression led to the identification of 170 genes in αCGRP-deficient mice, whose expression differed significantly from that in control WT mice. Interestingly, most of the genes with increased expression in mutant mice were part of or related to the family of immunoglobulins (**Suppl. Table 2**). However, this observation does not conclusively explain the impaired bone regeneration and most likely reflects the fact that excessive bone marrow including B-cells and plasma cells is found within the insufficient callus of mutant mice at this stage in bone healing. However, the genes with significantly lower expression compared to those in WT controls mice included regulators of bone formation (Adiponectin, *Adipoq*; Interleukin-1β, *Il1b*), bone remodeling (*CC-chemokine ligand 7, Ccl7*; Matrix metallopeptidase 13, *MMP13*; Mannose receptor C-type 1, *Mrc1*) and members of the PPARγ pathway (Adiponectin, *Adipoq*; adipocyte protein 2, *Fabp4*; Stearoyl-CoA desaturase, *Scd1*; Adipsin, *Cfd*), which were previously shown to differently affect bone homeostasis and bone regeneration, respectively (Fig. 8**a**) [44]. To ascertain whether these observations are potentially due to the stimulatory effect of nerve-derived αCGRP on callus cells, and whether they rely on the interaction of αCGRP with its receptor CRLR, bone-marrow cells were stimulated on day 2 of osteogenic differentiation with recombinant αCGRP and the CRLR antagonist olcegepant (BIBN) for 6 h and gene expression monitored. The results showed that short-term treatment with αCGRP resulted in a significantly increased expression of *Adipoq, MMP13, Mrc1, Il1b, Ccl7, Scd1*, and *Cfd,* which was blunted through simultaneous treatment with olcegepant in most of the genes tested (Fig. 8**b**). Similar results were obtained on day 5 in bone marrow-derived osteoblasts, which were stimulated short-term (6 h) or long-term (5 consecutive days) with αCGRP and olcegepant (**Suppl. Fig. 6**). At this intermediate stage of osteogenic differentiation, the CRLR-dependent induction of *MMP13* and *Il1b* was only present after short-term and not after long-term stimulation with αCGRP, whereas the opposite was observed with *Adipoq*. Together, these findings indicate that αCGRP activates a complex, time- and CRLR-dependent transcriptional response in osteoblast precursors within the fracture callus, which is essential for adequate bone regeneration.

## 4 Discussion

Given that impaired bone regeneration affects a significant proportion of patients with fractures, it is of high clinical and scientific importance to understand fracture healing at a mechanistic level and to identify mediators that essentially influence bone regeneration. Our study shows that CGRP and its receptor are differentially expressed in callus tissue during bone regeneration, and that αCGRP-deficient mice display profoundly impaired bone regeneration. This observation is explained by a striking reduction in the number of bone-forming osteoblasts in regenerating bone, resulting in a high rate of incomplete callus bridging and non-union. While a link between headache medication and fracture healing has not been explored in sufficient detail before, our results indicate that the molecular targets of novel anti-migraine drugs, which inhibit CGRP or the CGRP receptor, also function as physiologic and anabolic mediators in bone regeneration.

In our study, we provide direct *in vivo* evidence for an essential role of the neuropeptide αCGRP in fracture healing. Whereas we were previously able to demonstrate an important function of αCGRP as an osteoanabolic molecule maintaining bone formation in intact bone, resulting in osteopenia in αCGRP-deficient mice [11], the extent of regenerative impairment in these animals is indeed surprising. αCGRP-deficient mice did not only display a high rate of fracture non-union, but also a profound disturbance of callus organization on the cellular level. The fact that we observed increased CGRP levels following a femoral fracture in mice parallels clinical observations in patients with fracture of the proximal femur, in whom elevated CGRP levels have been reported for the first few days following injury[18,19]. Although we are unable to decipher a potential role of βCGRP based on the currently available CGRP antibodies, our findings show that CGRP and the CGRP receptor are expressed in regenerating bone at significant levels, especially during the early and intermediate stages of bone regeneration. Moreover, the current data suggests that the origin of systemically increased CGRP is indeed callus tissue, as an induction of *Calca* mRNA was only detected in regenerating bone and not in any other tissue studied. Most of CGRP immunoreactivity was detected either in the periosteum in close vicinity to the fracture callus or to invading blood vessels, both of which carry rich nerval innervation [10]. These findings clearly point towards a neuronal origin of αCGRP-induction during bone regeneration and highlight the importance of sensory innervation of fracture tissue during bone healing. In a recent study by Niedermair et al., it was suggested that αCGRP-deficiency alters M2 macrophage polarization without affecting callus maturation [21]. These divergent findings may be explained by the differences in the fracture model used. Neidermair et al. used intramedullary nailing with less periosteal disruption and absence of intramedullary callus expansion and the mice they used were older and ovariectomized. In contrast however, a study by Zhang et al., showed that implant-derived magnesium can promote bone formation through the activation of CGRP receptor signaling [22]. However, given that CGRP receptors do not only bind αCGRP but also possibly procalcitonin, a direct role of αCGRP in bone regeneration remained unclear [24,45]. Therefore, our study not only confirm the findings by Zhang et al. regarding the osteoanabolic effects of CGRP receptor signaling, but also for the first time, provides direct *in vivo* evidence for an essential role of αCGRP in fracture repair.

By employing genome-wide gene expression, we were able to identify more than 170 genes, which were differentially expressed in the callus of αCGRP-deficient and WT mice. In this regard, the induction of a large set of different immunoglobins (IG) was unexpected, however did not provide a conclusive explanation for the insufficient bone regeneration in mutant animals. Although cross-linking of activating Fcγ-receptors, which bind IG, has been shown by others to regulate osteoclast formation and activity in inflammatory and non-inflammatory conditions [46,47], a comparably crucial role of IG in regulating osteoblast differentiation and bone regeneration would indeed be surprising but cannot be excluded at this point. Importantly however, we identified several key regulators of bone formation and bone remodeling, which were expressed at significantly lower levels in the callus of mutant animals *in vivo,* and were positively regulated by CGRP in bone marrow-derived osteoblasts in a CGRP receptor-dependent manner *in vitro*. First, adiponectin was shown to stimulate osteoblast differentiation from bone mesenchymal stem cells and bone formation *in vitro* and *in vivo* [48], [49], [50]. Secondly, interleukin-1β functions not only as a crucial regulator of osteoclast activation during inflammatory conditions, but also promotes osteogenic differentiation and osteogenesis of osteoblast precursors [51,52]. Thirdly, CC-chemokine ligand 7 and the key macrophage phagocytic factor, mannose receptor C-type 1, are critically involved in the regulation of bone remodeling, especially with regards to osteoclast differentiation and matrix degradation, while MMP13 has been shown to be of pivotal importance in callus remodeling and fracture healing [53], [54], [55]. And finally, adiponectin, adipocyte protein 2, and adipsin are some of the most induced genes during osteogenic differentiation of primary osteoblasts *in vitro* [56] and, together with stearoyl-CoA desaturase, represent key mediators of the PPARγ pathway. During normal bone homeostasis, activation of PPARγ has been shown to result in an inhibition of bone formation, as differentiation of mesenchymal progenitors is directed towards adipogenesis [57]. However, our data imply that the induction of key mediators in PPARγ signaling is essential in the orchestration of callus remodeling and facilitation of adequate bone regeneration. This is consistent with recent findings by others, where inactivation of PPARγ signaling was shown to severely impair BMP2-induced osteogenesis and bone formation and, similar to αCGRP-deficient mice, resulted in bone non-union in a mouse femoral segmental defect model [44]. Moreover, CGRP signaling has been shown to be mediated through cAMP/PKA in osteoblasts, which is also essential for the activation of the PPARγ pathway [22,58,59].

While the precise cellular and molecular cascades involved in the interaction between CGRP and PPARγ signaling requires further mechanistic understanding, we believe that our findings are especially important from a clinical and health care point of view, particularly in orthopaedics and neurology. With regards to orthopaedics, the periosteal tissue containing vast amounts of sensory nerve fibres is often dissected intraoperatively in order to obtain better visibility and mobilization of fracture fragments for anatomic reduction. Given that excessive stripping of the periosteum is associated with a higher rate of impaired bone regeneration [60, 61], this may be explained, at least in part, by an insufficient release of αCGRP at the fracture site due, in part, to damaged nerves. With regards to neurology, all CGRP-targeted therapies tested for the acute treatment and prevention of migraine have consistently produced positive results to date, strongly supporting the evolving role of CGRP in migraine pathophysiology. Indeed, the orally administered CGRP receptor antagonist ubrogepant was shown to provide relief from acute migraine attacks [34,35], whereas monoclonal antibodies against either CGRP or CGRP receptor including erenumab, fremanezumab or galcanezumab, applied once monthly by subcutaneous injection, are particularly effective for a long-lasting prevention of episodic or chronic migraine [25]. The drugs currently being developed are well tolerated and exhibit an excellent safety profile free of warnings and precautions about adverse side effects aside from hypersensitivity and injection-site reactions [62]. In case of erenumab, pharmacology, pharmacokinetics, and toxicology studies showed no effects on cardiovascular risk, and specific staining of human tissues did not indicate any off-target binding [63]. In line with this, repeat-dose toxicology studies conducted in rats and cynomolgus monkeys showed no evidence of erenumab-mediated adverse toxicity. Although present clinical and nonclinical data obtained thus far suggest no safety concern, it must be emphasized that the risks of long- term blockade of CGRP signaling are currently not known, as with any new class of drug [64]. In this regard, our data indicates that caution needs to be taken, especially with long-lasting CGRP- and CGRP receptor-antibodies, as they may potentially interfere with bone regeneration, a particular aspect which has not yet been assessed in hitherto performed clinical studies to the best of our knowledge.

Based on these implications, our findings also indicate that CGRP agonism may be a promising approach to stimulate fracture healing in patients at risk for impaired bone regeneration. To date, the use of recombinant bone morphogenetic proteins remain the only FDA-approved pharmaceutical approach to stimulate fracture union, however their range of application is limited due to several potential detrimental adverse effects and the necessity of local application intraoperatively [1]. In the case of CGRP, selective agonists could in principle be administered systemically, significantly extending the possible range of treatment duration and frequency, and also providing a suitable option for improving non-surgical fracture treatment. Although the therapeutic potential of CGRP has been limited because of its peptide nature and short half-life, cardiovascular research has recently put forward injectable and long-lasting CGRP analogues which exert antihypertensive effects, attenuate cardiac failure and improve metabolic parameters in mice [65,66]. As such, it is of high clinical importance to examine whether these novel CGRP analogues are not only effective in metabolic and cardiac disease, but also in boosting bone regeneration, and whether potential benefits outweigh nociception known to be facilitated by CGRP.

In all, our study shows direct *in vivo* evidence for a pivotal role of αCGRP in bone regeneration, at least in mice. The data indicate that αCGRP is essentially required for adequate callus formation, affecting both bone-forming osteoblasts and bone-resorbing osteoclasts. Given that CGRP receptors are cell surface bound, making them excellent drug targets, selective CGRP agonism may prove a potential pharmacologic approach to treat impaired facture healing.

## Authors contributions

Study design: JK and ST. Study conducted: JA, AB, DJ, TY, SKF, PK, EO, MF, AH, ST. Data Collection: JA, AB, DJ, TY, PK, EO, MF, AH, ST. Data Analysis: JA, AB, TY, TS, ST, JK. Data interpretation: JA, AB, TY, MA, GND, ST, JK. Drafting and revision of manuscript: JA, AB, DJ, TY, SKF, PK, EO, MF, AH, FG, KHF, MA, TS, GND, ST, JK.

## Declarations of Competing Interest

All authors state that they have no conflict of interest.

## Appendix Supplementary materials

**Supplementary Figure 1.** (**a**) Schematic illustration of the applied osteotomy model with external fixator for stabilization in mice. (**b**) Representative \elsamp #x03BC;CT scanning of a femoral osteotomy. The region of interest (ROI) is indicated with the black box. Fracture cortices are indicated with dotted yellow lines. (**c**) Representative Movat (top) and TRAP (bottom) stainings of a femoral osteotomy. The black box shows the ROI employed for static and cellular histomorphometry, respectively. Fracture cortices are indicated with dotted black lines. Image, application 1

**Supplementary Figure 2.** Representative immunofluorescent stainings (merged) and individual channels of intact WT femur sections (diaphysis) using a CGRP-, endomucin- (Edm) and CD31-specific antibody. White dotted line indicates bone cortices. Image, application 2

**Supplementary Figure 3.** Representative, individual immunofluorescent stainings of WT callus sections 7, 14, 21 days after surgery using a CGRP-, Endomucin- (Edm) and CD31-specific antibody. white\elsamp #x00A0;\elsamp #x003D;\elsamp #x00A0;CGRP; green\elsamp #x00A0;\elsamp #x003D;\elsamp #x00A0;Edm, red\elsamp #x00A0;\elsamp #x003D;\elsamp #x00A0;CD31; blue\elsamp #x00A0;\elsamp #x003D;\elsamp #x00A0;DAPI. Image, application 3

**Supplementary Figure 4.** Representative, individual immunofluorescent stainings of WT callus sections 7, 14, 21 days after surgery using (**a**) CRLR and (**b**) RAMP1-specific antibodies. Red\elsamp #x00A0;\elsamp #x003D;\elsamp #x00A0;CRLR or RAMP1; blue\elsamp #x00A0;\elsamp #x003D;\elsamp #x00A0;DAPI. Image, application 4

**Supplementary Figure 5.** Negative control of osteocalcin staining in a murine femur bone. Image, application 5

**Supplementary Figure 6.** qRT-PCR expression analysis for the indicated genes in bone marrow-derived osteoblasts at day 5 of osteogenic differentiation with ascorbic acid and \elsamp #x03B2;-glycerophosphate, stimulated with CGRP (10^\elsamp #x2212;7^\elsamp #x00A0;M) and olcegepant (1\elsamp #x03BC;g/ml; BIBN) for 6\elsamp #x2009;h or 5 consecutive days as indicated. *n*\elsamp #x00A0;\elsamp #x003D;\elsamp #x00A0;4 independent cultures per group (two-way Anova followed by Tukey post-hoc test). Image, application 6

Image, application 7

Image, application 8

## References

[1] Russow G., Jahn D., Appelt J., Märdian S., Tsitsilonis S., Keller J (2018). Anabolic therapies in osteoporosis and bone regeneration. Int J Mol Sci.

[2] Tzioupis C., Giannoudis P.V. (2007). Prevalence of long-bone non-unions. Injury.

[3] Hak D.J., Fitzpatrick D., Bishop J.A., Marsh J.L., Tilp S., Schnettler R. (2014). Delayed union and non-unions: epidemiology, clinical issues, and financial aspects. Injury.

[4] Hankenson K.D., Gagne K., Shaughnessy M (2015). Extracellular signaling molecules to promote fracture healing and bone regeneration. Adv Drug Deliv Rev.

[5] Einhorn T.A., Gerstenfeld L.C (2015). Fracture healing: mechanisms and interventions. Nat Rev Rheumatol.

[6] Claes L., Recknagel S., Ignatius A (2012). Fracture healing under healthy and inflammatory conditions. Nat Rev Rheumatol.

[7] Emeson R.B., Hedjran F., Yeakley J.M., Guise J.W., Rosenfeld M.G (1989). Alternative production of calcitonin and CGRP mRNA is regulated at the calcitonin-specific splice acceptor. Nature.

[8] Emeson R.B., Yeakley J.M., Hedjran F., Merillat N., Lenz H.J., Rosenfeld M.G (1992). Posttranscriptional regulation of calcitonin/CGRP gene expression. Ann N Y Acad Sci.

[9] Naot D., Musson D.S., Cornish J (2019). The activity of peptides of the calcitonin family in bone. Physiol Rev.

[10] Irie K., Hara-Irie F., Ozawa H., Yajima T (2002). Calcitonin gene-related peptide (CGRP)-containing nerve fibers in bone tissue and their involvement in bone remodeling. Microsc Res Tech.

[11] Schinke T., Liese S., Priemel M., Haberland M., Schilling A.F., Catala-Lehnen P. (2004). Decreased bone formation and osteopenia in mice lacking alpha-calcitonin gene-related peptide. J Bone Miner Res.

[12] Ballica R., Valentijn K., Khachatryan A., Guerder S., Kapadia S., Gundberg C. (1999). Targeted expression of calcitonin gene-related peptide to osteoblasts increases bone density in mice. J Bone Miner Res.

[13] Mrak E., Guidobono F., Moro G., Fraschini G., Rubinacci A., Villa I (2010). Calcitonin gene-related peptide (CGRP) inhibits apoptosis in human osteoblasts by β-catenin stabilization. J Cell Physiol.

[14] Wang L., Shi X., Zhao R., Halloran B.P., Clark D.J., Jacobs C.R. (2010). Calcitonin-gene-related peptide stimulates stromal cell osteogenic differentiation and inhibits RANKL induced NF-kappaB activation, osteoclastogenesis and bone resorption. Bone.

[15] Tian G., Zhang G., Tan Y.H (2013). Calcitonin gene-related peptide stimulates BMP-2 expression and the differentiation of human osteoblast-like cells in vitro. Acta Pharmacol Sin.

[16] Zhou R., Yuan Z., Liu J (2016). Calcitonin gene-related peptide promotes the expression of osteoblastic genes and activates the WNT signal transduction pathway in bone marrow stromal stem cells. Mol Med Rep.

[17] Huebner A.K., Keller J., Catala-Lehnen P., Perkovic S., Streichert T., Emeson R.B. (2008). The role of calcitonin and alpha-calcitonin gene-related peptide in bone formation. Arch Biochem Biophys.

[18] Onuoha G.N., Alpar E.K. (2000). Elevation of plasma CGRP and SP levels in orthopedic patients with fracture neck of femur. Neuropeptides.

[19] Onuoha G.N. (2001). Circulating sensory peptide levels within 24h of human bone fracture. Peptides.

[20] Li J., Kreicbergs A., Bergström J., Stark A., Ahmed M (2007). Site-specific CGRP innervation coincides with bone formation during fracture healing and modeling: a study in rat angulated tibia. J Orthop Res.

[21] Niedermair T., Straub R.H., Brochhausen C., Grässel S (2020). Impact of the sensory and sympathetic nervous system on fracture healing in ovariectomized mice. Int J Mol Sci.

[22] Zhang Y., Xu J., Ruan Y.C., Yu M.K., O'Laughlin M., Wise H. (2016). Implant-derived magnesium induces local neuronal production of CGRP to improve bone-fracture healing in rats. Nat Med.

[23] Finlay D.B., Duffull S.B., Glass M (2020). 100 years of modelling ligand-receptor binding and response: a focus on GPCRs. Br J Pharmacol.

[24] Hay D.L., Garelja M.L., Poyner D.R., Walker C.S (2018). Update on the pharmacology of calcitonin/CGRP family of peptides: IUPHAR Review 25. Br J Pharmacol.

[25] Edvinsson L., Haanes K.A., Warfvinge K., Krause D.N (2018). CGRP as the target of new migraine therapies - successful translation from bench to clinic. Nat Rev Neurol.

[26] Goadsby P.J., Edvinsson L., Ekman R (1988). Release of vasoactive peptides in the extracerebral circulation of humans and the cat during activation of the trigeminovascular system. Ann Neurol.

[27] Goadsby P.J., Edvinsson L., Ekman R (1990). Vasoactive peptide release in the extracerebral circulation of humans during migraine headache. Ann Neurol.

[28] Gallai V., Sarchielli P., Floridi A., Franceschini M., Codini M., Glioti G. (1995). Vasoactive peptide levels in the plasma of young migraine patients with and without aura assessed both interictally and ictally. Cephalalgia.

[29] Buzzi M.G., Carter W.B., Shimizu T., Heath H., Moskowitz M.A (1991). Dihydroergotamine and sumatriptan attenuate levels of CGRP in plasma in rat superior sagittal sinus during electrical stimulation of the trigeminal ganglion. Neuropharmacology.

[30] Escott K.J., Beattie D.T., Connor H.E., Brain S.D (1995). Trigeminal ganglion stimulation increases facial skin blood flow in the rat: a major role for calcitonin gene-related peptide. Brain Res.

[31] MacDonald N.J., Butters L., O'Shaughnessy D.J., Riddell A.J., Rubin P.C (1989). A comparison of the effects of human alpha calcitonin gene-related peptide and glyceryl trinitrate on regional blood velocity in man. Br J Clin Pharmacol.

[32] Charles A., Pozo-Rosich P (2019). Targeting calcitonin gene-related peptide: a new era in migraine therapy. Lancet.

[33] Tepper S.J. (2018). History and review of anti-calcitonin gene-related peptide (CGRP) therapies: from translational research to treatment. Headache.

[34] Scott L.J (2020). Ubrogepant: first approval. Drugs.

[35] Lipton R.B., Dodick D.W., Ailani J., Lu K., Finnegan M., Szegedi A. (2019). Effect of ubrogepant vs placebo on pain and the most bothersome associated symptom in the acute treatment of migraine: the ACHIEVE II randomized clinical trial. JAMA.

[36] Ceriani C.E.J., Wilhour D.A., Silberstein S.D (2019). Novel medications for the treatment of migraine. Headache.

[37] Lu J.T., Son Y.J., Lee J., Jetton T.L., Shiota M., Moscoso L. (1999). Mice lacking alpha-calcitonin gene-related peptide exhibit normal cardiovascular regulation and neuromuscular development. Mol Cell Neurosci.

[38] Tsitsilonis S., Seemann R., Misch M., Wichlas F., Haas N.P., Schmidt-Bleek K. (2015). The effect of traumatic brain injury on bone healing: an experimental study in a novel in vivo animal model. Injury.

[39] Hildebrand T., Rüegsegger P. (1997). Quantification of bone microarchitecture with the structure model index. Comput Methods Biomech Biomed Engin.

[40] Bouxsein M.L., Boyd S.K., Christiansen B.A., Guldberg R.E., Jepsen K.J., Müller R (2010). Guidelines for assessment of bone microstructure in rodents using micro-computed tomography. J Bone Miner Res.

[41] Seemann R., Graef F., Garbe A., Keller J., Huang F., Duda G. (2018). Leptin-deficiency eradicates the positive effect of traumatic brain injury on bone healing: histological analyses in a combined trauma mouse model. J Musculoskelet Neuronal Interact.

[42] Dempster D.W., Compston J.E., Drezner M.K., Glorieux F.H., Kanis J.A., Malluche H. (2013). Standardized nomenclature, symbols, and units for bone histomorphometry: a 2012 update of the report of the ASBMR histomorphometry nomenclature committee. J Bone Miner Res.

[43] Keller J., Catala-Lehnen P., Huebner A.K., Jeschke A., Heckt T., Lueth A. (2014). Calcitonin controls bone formation by inhibiting the release of sphingosine 1-phosphate from osteoclasts. Nat Commun.

[44] Wang C., Tanjaya J., Shen J., Lee S., Bisht B., Pan H.C. (2019). Peroxisome proliferator-activated receptor-γ knockdown impairs bone morphogenetic protein-2-induced critical-size bone defect repair. Am J Pathol.

[45] Sexton P.M., Christopoulos G., Christopoulos A., Nylen E.S., Snider R.H., Becker K.L (2008). Procalcitonin has bioactivity at calcitonin receptor family complexes: potential mediator implications in sepsis. Crit Care Med.

[46] Negishi-Koga T., Gober H.J., Sumiya E., Komatsu N., Okamoto K., Sawa S. (2015). Immune complexes regulate bone metabolism through FcRγ signalling. Nat Commun.

[47] Seeling M., Nimmerjahn F. (2015). Unlocking the bone: fcγ-receptors and antibody glycosylation are keys to connecting bone homeostasis to humoral immunity. Ann Transl Med.

[48] Wang Y., Zhang X., Shao J., Liu H., Liu X., Luo E (2017). Adiponectin regulates BMSC osteogenic differentiation and osteogenesis through the Wnt/β-catenin pathway. Sci Rep.

[49] Lee H.W., Kim S.Y., Kim A.Y., Lee E.J., Choi J.Y., Kim J.B (2009). Adiponectin stimulates osteoblast differentiation through induction of COX2 in mesenchymal progenitor cells. Stem Cells.

[50] Williams G.A., Wang Y., Callon K.E., Watson M., Lin J.M., Lam J.B. (2009). In vitro and in vivo effects of adiponectin on bone. Endocrinology.

[51] Sonomoto K., Yamaoka K., Oshita K., Fukuyo S., Zhang X., Nakano K. (2012). Interleukin-1β induces differentiation of human mesenchymal stem cells into osteoblasts via the Wnt-5a/receptor tyrosine kinase-like orphan receptor 2 pathway. Arthritis Rheum.

[52] Mumme M., Scotti C., Papadimitropoulos A., Todorov A., Hoffmann W., Bocelli-Tyndall C. (2012). Interleukin-1β modulates endochondral ossification by human adult bone marrow stromal cells. Eur Cell Mater.

[53] Kosaki N., Takaishi H., Kamekura S., Kimura T., Okada Y., Minqi L. (2007). Impaired bone fracture healing in matrix metalloproteinase-13 deficient mice. Biochem Biophys Res Commun.

[54] Kitase Y., Lee S., Gluhak-Heinrich J., Johnson M.L., Harris S.E., Bonewald L.F (2014). CCL7 is a protective factor secreted by mechanically loaded osteocytes. J Dent Res.

[55] Sinder B.P., Zweifler L., Koh A.J., Michalski M.N., Hofbauer L.C., Aguirre J.I. (2017). Bone mass is compromised by the chemotherapeutic trabectedin in association with effects on osteoblasts and macrophage efferocytosis. J Bone Miner Res.

[56] Staines K.A., Zhu D., Farquharson C., MacRae V.E (2014). Identification of novel regulators of osteoblast matrix mineralization by time series transcriptional profiling. J Bone Miner Metab.

[57] Akune T., Ohba S., Kamekura S., Yamaguchi M., Chung U.I., Kubota N. (2004). PPARgamma insufficiency enhances osteogenesis through osteoblast formation from bone marrow progenitors. J Clin Invest.

[58] Kim S.P., Ha J.M., Yun S.J., Kim E.K., Chung S.W., Hong K.W. (2010). Transcriptional activation of peroxisome proliferator-activated receptor-gamma requires activation of both protein kinase A and Akt during adipocyte differentiation. Biochem Biophys Res Commun.

[59] Zou R., Xu G., Liu X.C., Han M., Jiang J.J., Huang Q. (2010). PPARgamma agonists inhibit TGF-beta-PKA signaling in glomerulosclerosis. Acta Pharmacol Sin.

[60] Oni O.O., Gregg P.J. (1990). The relative contribution of individual osseous circulations to diaphyseal cortical blood supply. J Orthop Trauma.

[61] Neagu T.P., Ţigliş M., Cocoloş I., Jecan C.R (2016). The relationship between periosteum and fracture healing. Rom J Morphol Embryol.

[62] Yuan H., Spare N.M., Silberstein S.D (2019). Targeting CGRP for the prevention of migraine and cluster headache: a narrative review. Headache.

[63] Bussiere J.L., Davies R., Dean C., Xu C., Kim K.H., Vargas H.M. (2019). Nonclinical safety evaluation of erenumab, a CGRP receptor inhibitor for the prevention of migraine. Regul Toxicol Pharmacol.

[64] Deen M., Correnti E., Kamm K., Kelderman T., Papetti L., Rubio-Beltrán E. (2017). Blocking CGRP in migraine patients - a review of pros and cons. J Headache Pain.

[65] Aubdool A.A., Thakore P., Argunhan F., Smillie S.J., Schnelle M., Srivastava S. (2017). A novel α-calcitonin gene-related peptide analogue protects against end-organ damage in experimental hypertension, cardiac hypertrophy, and heart failure. Circulation.

[66] Nilsson C., Hansen T.K., Rosenquist C., Hartmann B., Kodra J.T., Lau J.F. (2016). Long acting analogue of the calcitonin gene-related peptide induces positive metabolic effects and secretion of the glucagon-like peptide-1. Eur J Pharmacol.

