# Supplementary figures and images for: The neuropeptide calcitonin gene-related peptide alpha is essential for bone healing
Source: eBioMedicine. 2020 Aug 24;59:102970. doi: 10.1016/j.ebiom.2020.102970 (PMC7452713; doi:10.1016/j.ebiom.2020.102970)

# Supplementary Figure 1

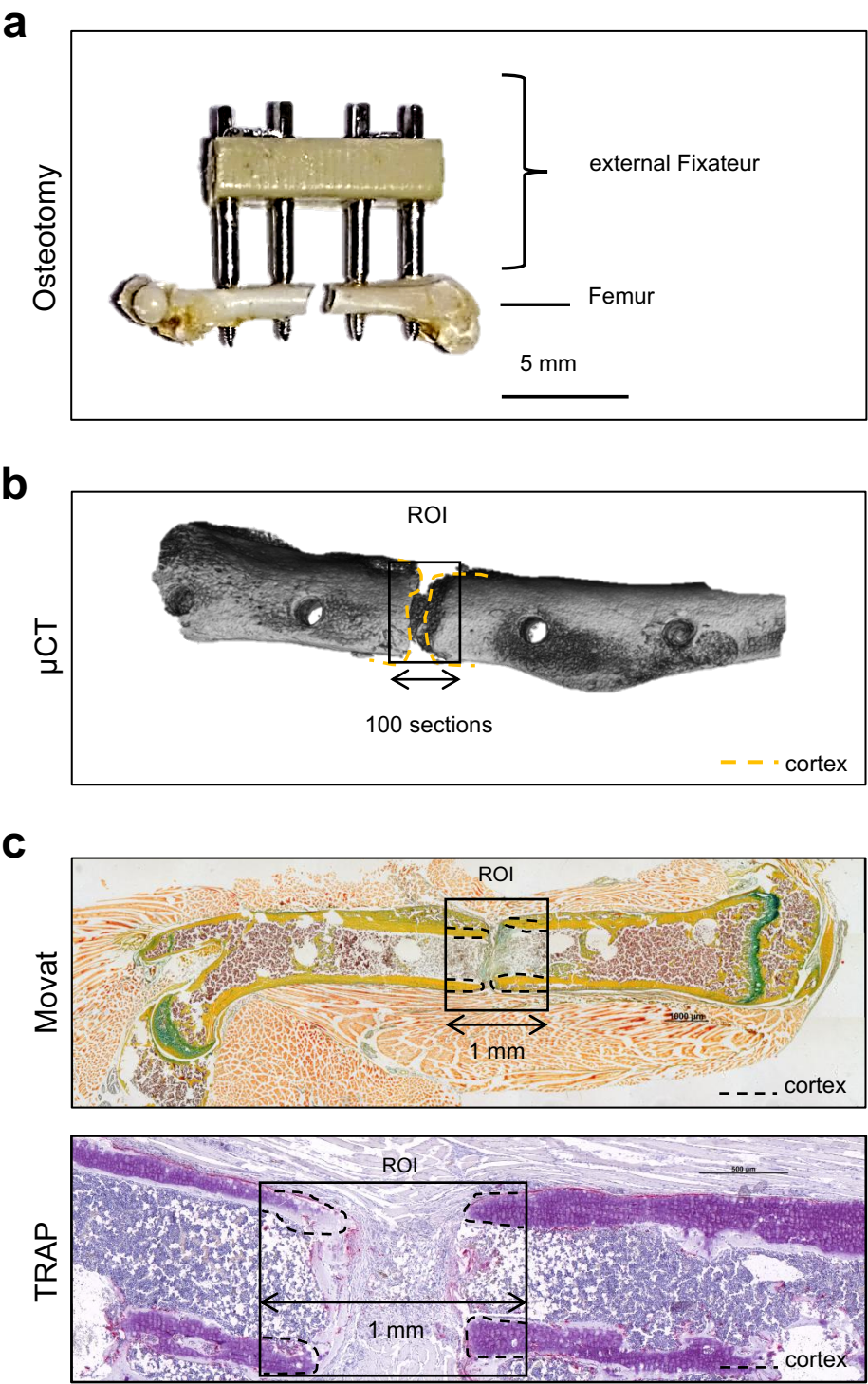

Supplement: Supplementary file 1 — Supplementary Figure 1. (a) Schematic illustration of the applied osteotomy model with external fixator for stabilization in mice. (b) Representative \elsamp #x03BC;CT scanning of a femoral osteotomy. The region of interest (ROI) is indicated with the black box. Fracture cortices are indicated with dotted yellow lines. (c) Representative Movat (top) and TRAP (bottom) stainings of a femoral osteotomy. The black box shows the ROI employed for static and cellular histomorphometry, respectively. Fracture cortices are indicated with dotted black lines. [file mmc1.pdf]

# Supplementary Figure 2

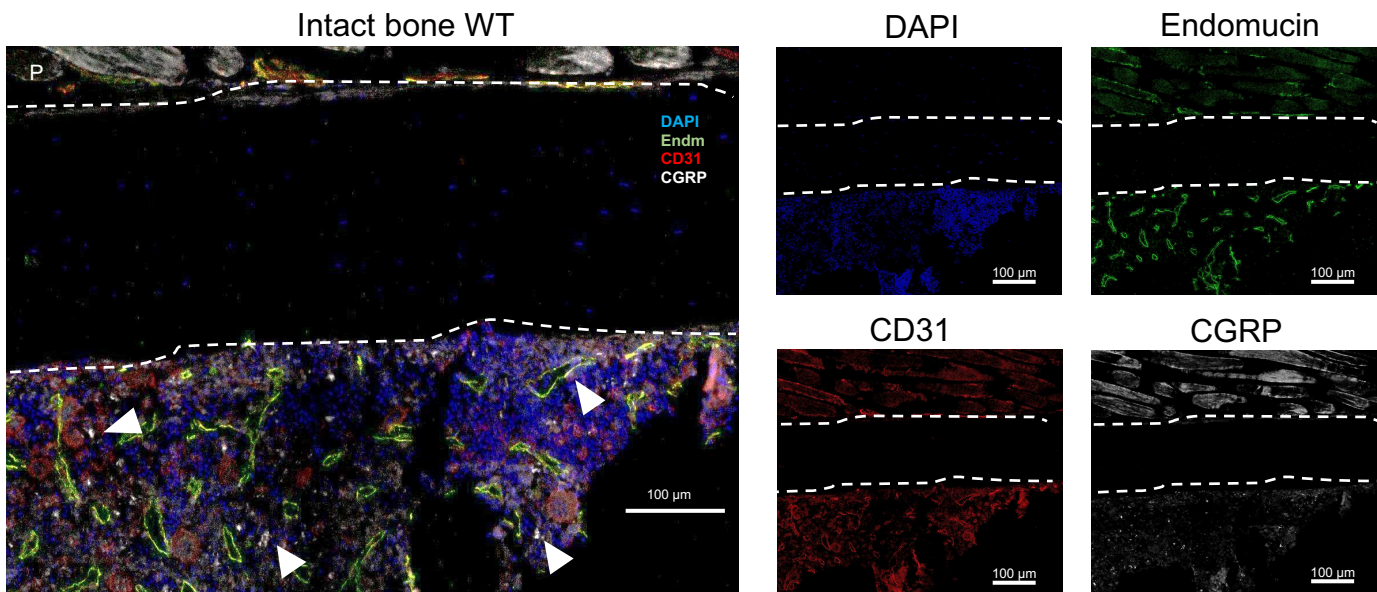

Supplement: Supplementary file 2 — Supplementary Figure 2. Representative immunofluorescent stainings (merged) and individual channels of intact WT femur sections (diaphysis) using a CGRP-, endomucin- (Edm) and CD31-specific antibody. White dotted line indicates bone cortices. [file mmc2.pdf]

# Supplementary Figure 3

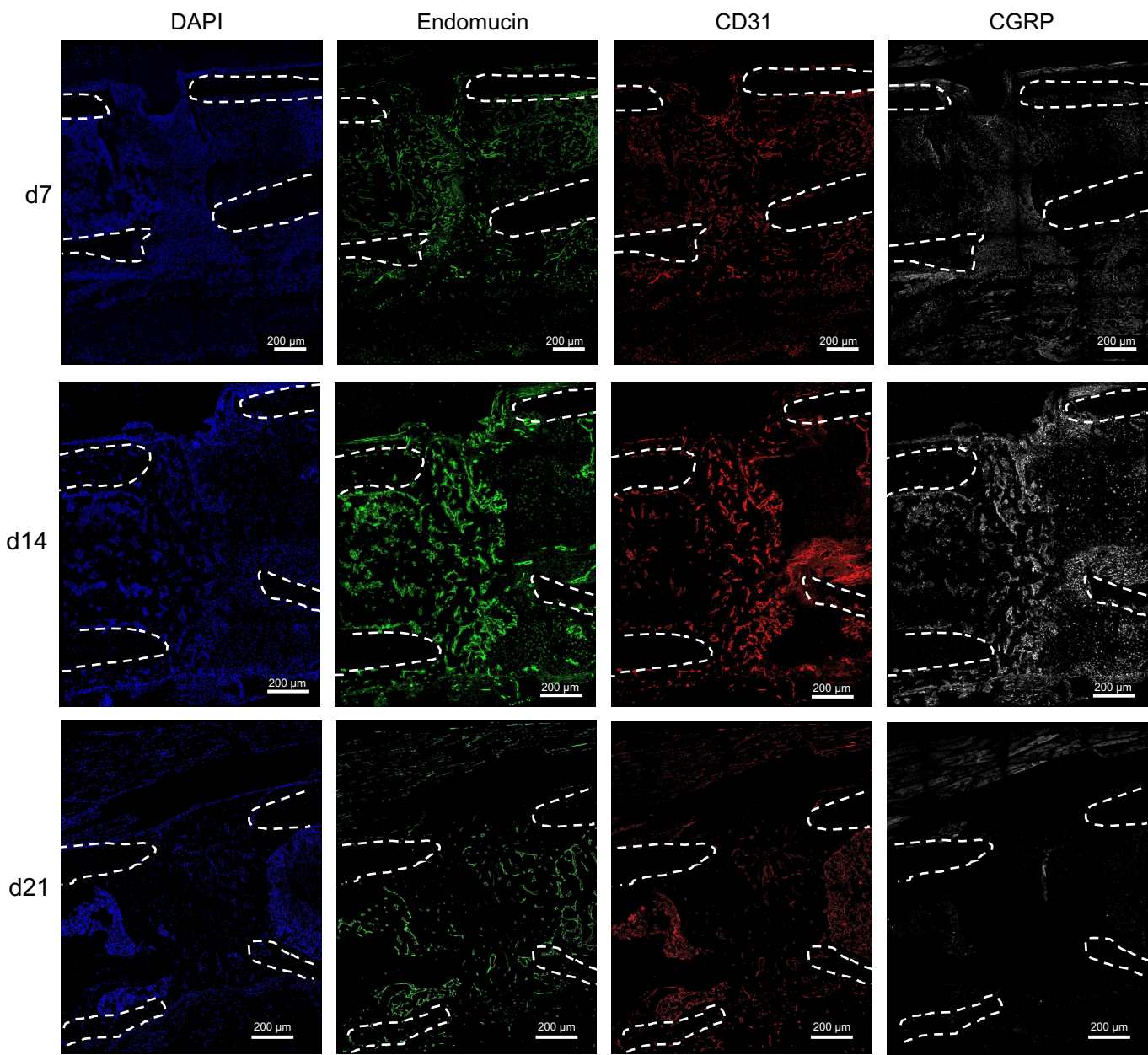

Supplement: Supplementary file 3 — Supplementary Figure 3. Representative, individual immunofluorescent stainings of WT callus sections 7, 14, 21 days after surgery using a CGRP-, Endomucin- (Edm) and CD31-specific antibody. white\elsamp #x00A0;\elsamp #x003D;\elsamp #x00A0;CGRP; green\elsamp #x00A0;\elsamp #x003D;\elsamp #x00A0;Edm, red\elsamp #x00A0;\elsamp #x003D;\elsamp #x00A0;CD31; blue\elsamp #x00A0;\elsamp #x003D;\elsamp #x00A0;DAPI. [file mmc3.pdf]

Supplementary Figure 4

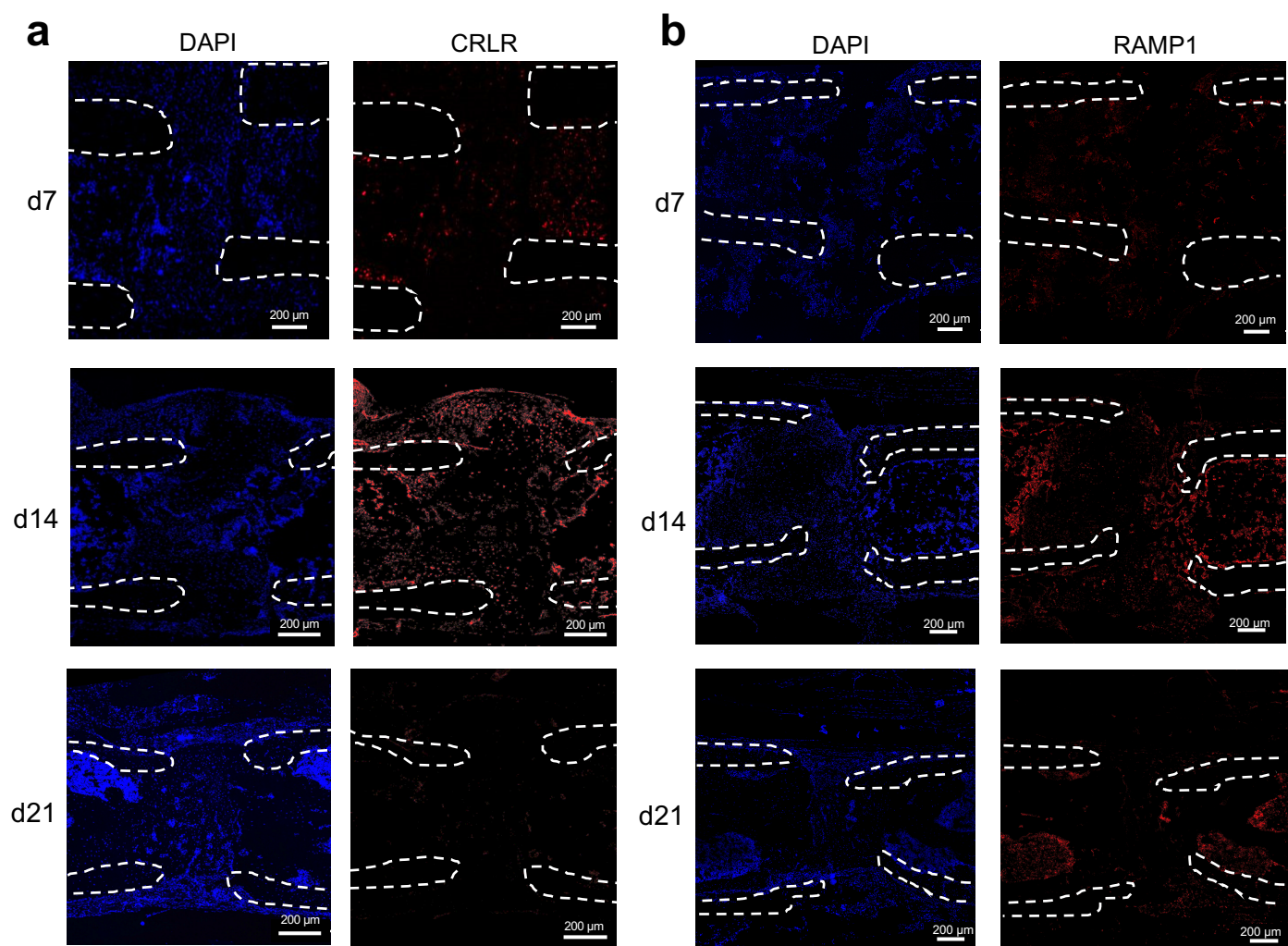

Supplement: Supplementary file 4 — Supplementary Figure 4. Representative, individual immunofluorescent stainings of WT callus sections 7, 14, 21 days after surgery using (a) CRLR and (b) RAMP1-specific antibodies. Red\elsamp #x00A0;\elsamp #x003D;\elsamp #x00A0;CRLR or RAMP1; blue\elsamp #x00A0;\elsamp #x003D;\elsamp #x00A0;DAPI. [file mmc4.pdf]

Supplementary Figure 5

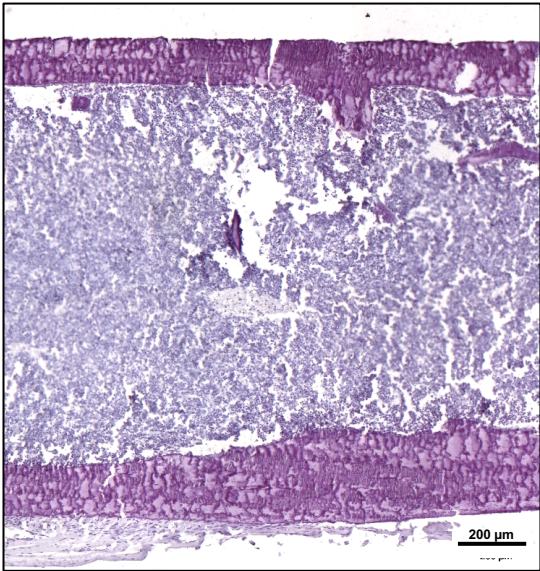

Supplement: Supplementary file 5 — Supplementary Figure 5. Negative control of osteocalcin staining in a murine femur bone. [file mmc5.pdf]

# Supplementary Figure 6

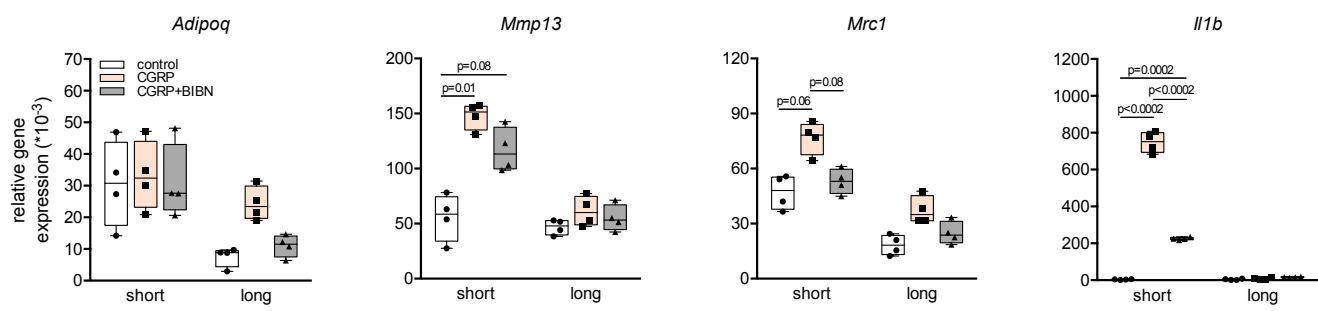

Supplement: Supplementary file 6 — Supplementary Figure 6. qRT-PCR expression analysis for the indicated genes in bone marrow-derived osteoblasts at day 5 of osteogenic differentiation with ascorbic acid and \elsamp #x03B2;-glycerophosphate, stimulated with CGRP (10\elsamp #x2212;7\elsamp #x00A0;M) and olcegepant (1\elsamp #x03BC;g/ml; BIBN) for 6\elsamp #x2009;h or 5 consecutive days as indicated. n\elsamp #x00A0;\elsamp #x003D;\elsamp #x00A0;4 independent cultures per group (two-way Anova followed by Tukey post-hoc test). [file mmc6.pdf]
