# Supplementary material for: The neuropeptide calcitonin gene-related peptide alpha is essential for bone healing
Source: eBioMedicine. 2020 Aug 24;59:102970. doi: 10.1016/j.ebiom.2020.102970 (PMC7452713; doi:10.1016/j.ebiom.2020.102970)
Supplement: Supplementary file 7 [file mmc7.docx]

**Supplementary Table 1.** Study design and number of animals employed

| **Genotype** | **Serum analysis/**  **callus qRT-PCR gene expression** | **μCT/histology/**  **immunohistochemistry/**  **immunofluorescence** | **Microarray** | ***In vitro***  **experiments** | **Total** |
| --- | --- | --- | --- | --- | --- |
| **WT (C57Bl/6J)** | n=6/time point (day 3, 7, 14)  total: 18 mice | n=6/time point (day 7, 14, 21)  total: 18 mice | n=3/time point (day 7)  total: 3 mice | total: 20 mice | **59** |
| **αCGRP^-/-^ (C57Bl/6J)** | - | n=6/time point (day 7, 14, 21)  total: 18 mice | n=3/time point (day 7)  total: 3 mice | total: 10 mice | **31** |
