# Supplementary material for: The neuropeptide calcitonin gene-related peptide alpha is essential for bone healing
Source: eBioMedicine. 2020 Aug 24;59:102970. doi: 10.1016/j.ebiom.2020.102970 (PMC7452713; doi:10.1016/j.ebiom.2020.102970)
Supplement: Supplementary file 8 [file mmc8.docx]

**Supplementary Table 2.** List of significantly induced genes in the callus at day 7 of bone regeneration (αCGRP-deficient mice vs WT controls; n = 3 per group) as determined by gene array hybridization.

| **CGRP^-/-^ Avg (log2)** | **CGRP^+/+^ Avg (log2)** | **Fold Change** | **p-val** | **Gene** |
| --- | --- | --- | --- | --- |
| 8.97 | 5.29 | 12.83 | 0.0365 | Myh7 |
| 11.21 | 7.64 | 11.88 | 0.0001 | Igkv1-110 |
| 10.46 | 6.97 | 11.27 | 0.0022 | Ighv1-80 |
| 9.38 | 6.11 | 9.69 | 0.0232 | Igkv4-57 |
| 7.68 | 4.41 | 9.63 | 0.0119 | Mir669b |
| 10.38 | 7.24 | 8.77 | 0.0116 | Igkv4-59 |
| 10.36 | 7.23 | 8.72 | 0.0291 | Igkv4-54 |
| 9.41 | 6.34 | 8.41 | 0.0279 | Igkv4-62 |
| 8.17 | 5.14 | 8.12 | 0.0020 | Ighv1-64 |
| 9.26 | 6.35 | 7.48 | 0.0008 | Ighv1-73 |
| 15.62 | 12.9 | 6.58 | 0.0040 | Ighv1-12 |
| 8.5 | 5.88 | 6.13 | 0.0270 | Igkv4-55 |
| 10.01 | 7.4 | 6.1 | 0.0361 | Igkv4-61 |
| 7.28 | 4.8 | 5.56 | 0.0002 | Igkv5-43 |
| 8.54 | 6.07 | 5.54 | 0.0252 | Igkv4-80 |
| 8.9 | 6.46 | 5.44 | 0.0339 | Igkv4-72 |
| 6.66 | 4.23 | 5.41 | 0.0015 | Igkv5-48 |
| 9.88 | 7.48 | 5.29 | 0.0431 | Igkv4-71 |
| 5.41 | 3.03 | 5.21 | 0.0031 | Ighv1-12 |
| 16.98 | 14.66 | 5.01 | 0.0217 | C1qtnf3 |
| 9.3 | 7.04 | 4.76 | 0.0089 | Ighv1-34 |
| 9.52 | 7.29 | 4.69 | 0.0003 | Igkv1-117; Igkv1-122 |
| 8.35 | 6.22 | 4.4 | 0.0018 | Ighv1-83 |
| 9.77 | 7.67 | 4.27 | 0.0024 | Ighv1-5 |
| 7.62 | 5.63 | 3.98 | 0.0274 | Igkv4-86 |
| 7.46 | 5.48 | 3.95 | 0.0048 | Ighv1-31 |
| 6.63 | 4.69 | 3.83 | 0.0263 | Ighv14-2 |
| 5.96 | 4.04 | 3.8 | 0.0072 | Ighv1-81 |
| 9.33 | 7.43 | 3.71 | 0.0210 | Igkv4-63 |
| 8.31 | 6.43 | 3.69 | 0.0008 | Ighv1-70 |
| 6.46 | 4.57 | 3.69 | 0.0148 | Ighv1-62-1 |
| 7.06 | 5.18 | 3.67 | 0.0142 | Igkv4-69 |
| 7.38 | 5.53 | 3.61 | 0.0057 | Ighv1-43 |
| 6.48 | 4.63 | 3.6 | 0.0076 | Igkv3-2 |
| 7.47 | 5.65 | 3.53 | 0.0218 | Ighv1-42 |
| 9.02 | 7.2 | 3.53 | 0.0024 | Ighv1-14 |
| 7.7 | 5.88 | 3.53 | 0.0484 | Ighv1-82 |
| 9.36 | 7.56 | 3.49 | 0.0077 | Ighv1-55 |
| 8.51 | 6.73 | 3.45 | 0.0011 | Ighv1-56 |
| 7.38 | 5.6 | 3.42 | 0.0077 | Ighv6-6 |
| 6.04 | 4.27 | 3.41 | 0.0198 | Ighv1-22 |
| 7.79 | 6.05 | 3.34 | 0.0031 | Ighv3-6 |
| 6.13 | 4.39 | 3.32 | 0.0080 | Igkv8-27 |
| 8.5 | 6.77 | 3.32 | 0.0352 | Igkv10-96; Igkv10-94 |
| 8.16 | 6.43 | 3.31 | 0.0175 | Gm4242 |
| 13.99 | 12.27 | 3.3 | 0.0088 | Thbs4 |
| 11.43 | 9.71 | 3.3 | 0.0360 | Gm11505; RP23-449P23.6 |
| 6.25 | 4.54 | 3.28 | 0.0216 | Igkv1-99 |
| 7.2 | 5.56 | 3.12 | 0.0021 | Ighv1-23 |
| 7.74 | 6.12 | 3.09 | 0.0038 | Ighv1-75 |
| 8.53 | 6.93 | 3.03 | 0.0073 | Ighv1-26 |
| 9.56 | 7.96 | 3.03 | 0.0025 | Tnnc1 |
| 7.32 | 5.75 | 2.98 | 0.0302 | Ighv1-74; Gm16710 |
| 6.18 | 4.61 | 2.97 | 0.0028 | Ighv1-79 |
| 6.62 | 5.08 | 2.91 | 0.0127 | Ighv1-85 |
| 5.34 | 3.81 | 2.89 | 0.0135 | Igkv3-1 |
| 6.82 | 5.29 | 2.89 | 0.0003 | Ighg2b |
| 6.76 | 5.24 | 2.88 | 0.0195 | Ighv1-19 |
| 7.2 | 5.68 | 2.87 | 0.0032 | Igkv8-30; Igkv3-7; Igkj1; Igkj2; Igkj4; Igkj5; Igkc; Igkv8-26; Igkv6-17; Igkv3-12; Igkv3-9; Igkv3-5 |
| 6.06 | 4.57 | 2.81 | 0.0287 | Igkv4-68 |
| 7.18 | 5.71 | 2.77 | 0.0004 | Jchain |
| 8.63 | 7.17 | 2.74 | 0.0114 | Ighv6-3 |
| 6.15 | 4.71 | 2.71 | 0.0115 | Ighv1-15 |
| 7.04 | 5.62 | 2.68 | 0.0002 | Ighv1-86 |
| 6.39 | 4.99 | 2.63 | 0.0326 | Olfml1 |
| 5.42 | 4.06 | 2.56 | 0.0005 | Ighv1-11 |
| 5.49 | 4.13 | 2.56 | 0.0494 | Myl2 |
| 5.56 | 4.2 | 2.56 | 0.0041 | Igkv3-10 |
| 4.38 | 3.06 | 2.5 | 0.0001 | Ighv1-19-1 |
| 7.19 | 5.87 | 2.49 | 0.0031 | Ighv1-9 |
| 4.92 | 3.61 | 2.47 | 0.0477 | 2310075C17Rik; RP23-152P11.3 |
| 8.27 | 6.98 | 2.44 | 0.0200 | Ighv1-69 |
| 6.68 | 5.43 | 2.37 | 0.0120 | Igkv6-25 |
| 4.75 | 3.5 | 2.37 | 0.0103 | Gm25382 |
| 6.45 | 5.21 | 2.36 | 0.0265 | Igkv4-57-1 |
| 6.75 | 5.51 | 2.36 | 0.0115 | Ighv1-77 |
| 5.49 | 4.25 | 2.35 | 0.0323 | Ighv1-39 |
| 6.3 | 5.08 | 2.33 | 0.0179 | Igkv4-56 |
| 7.13 | 5.92 | 2.31 | 0.0004 | Igkv6-15 |
| 6.11 | 4.91 | 2.31 | 0.0152 | Ighv1-18 |
| 8.6 | 7.4 | 2.3 | 0.0050 | Tnnt1 |
| 9.38 | 8.19 | 2.29 | 0.0303 | Ighv1-53 |
| 6.83 | 5.65 | 2.27 | 0.0457 | Ighv1-52 |
| 7.04 | 5.88 | 2.24 | 0.0123 | Ighv1-66 |
| 8.89 | 7.73 | 2.23 | 0.0454 | Atp1b1 |
| 8 | 6.86 | 2.2 | 0.0217 | Pamr1 |
| 10.04 | 8.91 | 2.19 | 0.0186 | LOC100861805 |
| 8.44 | 7.31 | 2.18 | 0.0081 | Igkv15-103 |
| 5.1 | 3.98 | 2.18 | 0.0008 | Igkv5-45 |
| 6.14 | 5.06 | 2.12 | 0.0052 | Ighv1-63 |
| 9.64 | 8.56 | 2.11 | 0.0242 | Angptl1 |
| 6.53 | 5.48 | 2.07 | 0.0118 | Ighv1-36 |
| 7.44 | 6.4 | 2.05 | 0.0461 | Igkv4-70 |
| 5.6 | 4.57 | 2.05 | 0.0198 | Ighv14-1 |
